# Beyond not bad or just okay: social predictors of young adults’ wellbeing and functioning (a TRAILS study)

**DOI:** 10.1017/S0033291718001976

**Published:** 2018-09-19

**Authors:** J. S. Richards, C. A. Hartman, B. F. Jeronimus, J. Ormel, S. A. Reijneveld, R. Veenstra, F. C. Verhulst, W. A. M. Vollebergh, A. J. Oldehinkel

**Affiliations:** 1 University of Groningen, University Medical Center Groningen, Department of Psychiatry, Interdisciplinary Center Psychopathology and Emotion Regulation (ICPE), Groningen, The Netherlands; 2 University of Groningen, Department of Developmental Psychology, Faculty of Social and Behavioural Sciences, Groningen, The Netherlands; 3 University of Groningen, University Medical Center Groningen, Department of Health Sciences, Groningen, The Netherlands; 4 University of Groningen, Department of Sociology, Interuniversity Center for Social Science Theory and Methodology (ICS), Groningen, The Netherlands; 5 Erasmus University Medical Center Rotterdam, Department of Child Psychiatry/Psychology, Rotterdam, The Netherlands; 6 Child and Adolescent Mental Health Center, Mental Health Services, Capital Region of Denmark, Copenhagen, Denmark; 7 University of Copenhagen, Department of Clinical Medicine, Faculty of Health and Medical Sciences, Copenhagen, Denmark; 8 Utrecht University, Department of Interdisciplinary Social Sciences, Utrecht, The Netherlands

**Keywords:** Adolescence, family relations, multidimensional functioning, peer relations, young adulthood

## Abstract

**Background:**

Various childhood social experiences have been reported to predict adult outcomes. However, it is unclear how different social contexts may influence each other's effects in the long run. This study examined the joint contribution of adolescent family and peer experiences to young adult wellbeing and functioning.

**Methods:**

Participants came from the TRacking Adolescents’ Individual Lives Survey (TRAILS) study (*n* = 2230). We measured family and peer relations at ages 11 and 16 (i.e. family functioning, perceived parenting, peer status, peer relationship quality), and functioning as the combination of subjective wellbeing, physical and mental health, and socio-academic functioning at age 22. Using structural equation modelling, overall functioning was indicated by two latent variables for positive and negative functioning. Positive, negative and overall functioning at young adulthood were regressed on adolescent family experiences, peer experiences and interactions between the two.

**Results:**

Family experiences during early and mid-adolescence were most predictive for later functioning; peer experiences did not independently predict functioning. Interactions between family and peer experiences showed that both protective and risk factors can have context-dependent effects, being exacerbated or overshadowed by negative experiences or buffered by positive experiences in other contexts. Overall the effect sizes were modest at best.

**Conclusions:**

Adolescent family relations as well as the interplay with peer experiences predict young adult functioning. This emphasizes the importance of considering the relative effects of one context in relation to the other.

The social environment plays a crucial role in child development. Parents are the first key figures in children's lives. Later, parental influences remain substantial, but become intertwined with peer influences. Drawn from different theoretical frameworks (e.g. attachment theory, social dynamics and social network theory), these social relations are believed to shape children's emotional and behavioural development through mechanisms including social support, social influence, social engagement and attachment, and access to resources (Berkman *et al*., 2000; Smith and Christakis, 2008). For example, attachment theory postulates that having secure attachments provides a safe basis, a sense of security and self-esteem, which are regarded to be fundamental for healthy emotional development (Bowlby, 1973). Indeed, adolescents attaining good quality relationships with parents and friends tend to be generally better adjusted than those with lower quality relations (e.g. Umberson *et al*., 2010). Early social contexts are also predictive of future adult adjustment. Longitudinal studies have shown that family experiences in adolescence predict multiple facets of functioning in adulthood, including physical and mental health, wellbeing and academic achievement (e.g. Korkeila *et al*., 2004; Paradis *et al*., 2009; Huppert *et al*., 2010; Harding *et al*., 2015). Similar findings have been reported for peer relations, which have been linked to mental health, overall disease risk, (un)employment and academic achievement in adulthood (e.g. Gest *et al*., 2006; Almquist and Brännström, 2014; Sakyi *et al*., 2015).

Experiences from different social contexts do not act in isolation; ecological and social systems theories (Bronfenbrenner, 1979; Hartup, 1989) posit that family and peer environments are interconnected systems, with influences of one system interacting with the other. For example, in line with the ‘dual-hit’ hypothesis, risk effects may be especially salient when negative experiences are present in both family and peer contexts, compared with problems in only one domain (Hazel *et al*., 2014). Alternatively, the stress-buffering model (Cohen and Wills, 1985; Rutter, 1985) suggests that supportive social environments can buffer stressful experiences or risk factors, such as problematic family or peer relations.

Prior research has shown that family and peer relations can both attenuate and exacerbate each other's effects (Gauze *et al*., 1996; Lansford *et al*., 2003; Gaertner *et al*., 2010; Sentse *et al*., 2010; Véronneau and Dishion, 2010; Trudeau *et al*., 2012). However, studies focusing on the effects of adolescent family and peer interplay into adulthood are scarce (see for exceptions Morojele and Brook, 2001; Pesola *et al*., 2015), leaving it unclear whether and how such cross-context interactions may influence each other's effects in the long run. In addition, previous studies investigating cross-context interactions have often focused on certain aspects of social relations measured once or twice within a narrow developmental timeframe. Yet, the relative contribution of family and peer effects may change across adolescence, when the perception of parents as a primary source of support shifts to that of peers (Furman and Buhrmester, 1992). It is therefore highly relevant and informative to assess the effects of these experiences during multiple time points. Moreover, most research on social influences conducted so far has focused on either positive or negative functional outcomes, and often only on specific aspects thereof. This does not capture the complex and multidimensional nature of human functioning. In studies focusing on psychopathology, the absence of mental health problems is often considered as a positive outcome. However, health is more than the absence of disease or illness, rather it is ‘a state of complete physical, mental and social wellbeing’ (World Health Organization, 2006). Similarly, low positive functioning or wellbeing does not imply the presence of a mental disorder. Hence, assessing one aspect of functioning, such as mental health, does not necessarily give insight into functioning in other domains and, ideally, outcome measures should capture both positive and negative dimensions of several domains of functioning in order to gain insight into an individual's overall functional state (e.g. Westerhof and Keyes, 2010).

This study investigated which family and peer relations during early and middle adolescence (ages 11 and 16) predicted young adult functioning (age 22) in a prospective cohort study of Dutch adolescents. We expanded upon prior research by (a) not only considering long-term main effects, but also the interplay between family and peer environments, (b) including a comprehensive assessment of family and peer relations during two time points in adolescence, (c) adopting a multidimensional approach on functioning – encompassing subjective wellbeing, physical and mental health and socio-academic functioning – and (d) moving beyond a ‘not bad or just OK’ approach by including both positive and negative sides of health and functioning. To our knowledge, this is one of the first studies to focus on the long-term effects of cross-context interactions on multidimensional functioning in young adulthood.

## Method

### Participants and procedure

Participants were selected from the Dutch prospective cohort study TRAILS (TRacking Adolescents’ Individual Lives Survey), which involves bi- or triennial follow-up measurements. Detailed descriptions of TRAILS can be found in previous reports (Ormel *et al*., 2012; Oldehinkel *et al*., 2015). Adolescents were recruited from five municipalities in the north of the Netherlands, including both urban and rural areas. Six assessment waves have been completed to date. The present study focused on the first (T1; 2001–2002), third (T3; 2005–2007) and fifth (T5; 2012–2013) wave. A total of 2230 adolescents enrolled at T1 (response rate 76%, mean age 11.1 years, 51% female). The response rates at T3 and T5 were, respectively, 81% (*n* = 1816, mean age 16.3, 52% female) and 80% (*n* = 1778, mean age 22.3, 53% female). The study was approved by the Dutch Central Committee on Research Involving Human Subjects (CCMO). Participants were treated in accordance with APA ethical standards and the Declaration of Helsinki, and all measurements were carried out with their adequate understanding and written consent.

### Measures

Below, a brief description of the included measures is given, while more detailed information, including references for their validation, is provided in the online Supplementary material. An overview of the measures used in this study including information on informant, number of items and internal consistency, can be found in Table 1 (the scale scores represent the mean item score).

**Table 1.** Descriptive statistics of study variables

	*N*	*M*/%	s.d.	Min	Max	Informant	Items	Item scale	*α*
Sex (male)	1098	49.20							
Age T1	2230	11.11	0.56	10.01	12.58				
Age T3	1819	16.28	0.71	14.69	18.69				
Age T5	1778	22.29	0.65	21.03	24.10				
Family environment T1									
Family dysfunction	2043	1.77	0.36	1.00	3.67	P	12	1–4^a^	0.87
Parental warmth	2207	3.21	0.50	1.17	4.00	A	18	1–4^b^	0.91/0.91^c^
Parental rejection	2206	1.48	0.31	1.00	3.47	A	17	1–4^b^	0.84/0.83^c^
Parental overprotection	2206	1.86	0.38	1.00	3.50	A	12	1–4^b^	0.70/0.71^c^
Peer environment T1									
Peer status	2165	3.10	0.69	1.00	5.00	A	4	1–5^b^	0.70
Peer affection	2168	3.99	0.48	1.00	5.00	A	8	1–5^b^	0.91
Family environment T3									
Family dysfunction	1503	1.65	0.40	1.00	3.50	P	12	1–4^a^	0.85
Parental control	1650	2.20	0.94	0.00	4.00	A	5	0–4^b^	0.84/0.79^c^
Parental angry outbursts	1645	1.10	0.77	0.00	4.00	A	3	0–4^b^	0.78/0.76^c^
Parental guilt inducing	1638	0.29	0.50	0.00	4.00	A	3	0–4^b^	0.77/0.74^c^
Parental problem solving	1652	2.25	0.84	0.00	4.00	A	4	0–4^b^	0.77/0.77^c^
Peer environment T3									
Peer support	1489	3.76	0.91	1.00	5.00	A	1	1–5^b^	–
Practical help peers	1493	3.17	1.06	1.00	5.00	A	1	1–5^b^	–
Peer fights	1493	1.45	0.49	1.00	5.00	A	1	1–5^b^	–
Multidimensional functioning T5									
Positive functioning T5									
Physical health	1498	3.19	0.81	1.00	4.00	A	1	1–4^d^	–
Positive affect	1497	3.51	0.54	1.00	5.00	A	10	1–5^b^	0.83
Happiness	1497	7.48	1.44	1.00	10.00	A	1	1–10^e^	–
Satisfaction	1512	6.54	1.44	0.00	9.00	A	6	0–9^f^	0.63
Positive social functioning	1605	1.67	0.39	0.00	2.00	P	4	0–2^g^	0.55
Personal achievement	1607	1.48	0.37	0.00	2.00	P	5	0–2^g^	0.63
Educational attainment	1524	3.72	0.91	1.00	5.00	A	2	1–5^h^	–
Daily occupation study/work	1523	2.79	0.57	1.00	3.00	A	4	1–3^i^	–
Negative functioning T5									
Negative affect	1497	2.06	0.65	1.00	4.70	A	10	1–5^b^	0.89
Affective problems	1745	0.33	0.27	0.00	1.97	A + P	21	0–2^g^	0.89/0.88^j^
Attention problems	1746	0.37	0.29	0.00	1.77	A + P	13	0–2^g^	0.82/0.88^j^
Antisocial personality problems	1746	0.14	0.02	0.00	1.45	A + P	20	0–2^g^	0.73/0.84^j^
Avoidant personality problems	1745	0.27	0.09	0.00	1.86	A + P	7	0–2^g^	0.80/0.80^j^

A, adolescent; P, parent.

a Strongly agree–strongly disagree.

b Never–(almost) always/very often.

c Reliability questionnaire about father/mother.

d Bad–good.

e Very unhappy–very happy.

f Very unsatisfied–very satisfied.

g Not true–very or often true.

h Primary–university.

i No occupation–full-time occupation.

j Reliability adolescent/parent report.

#### Family functioning and parenting (T1 and T3)

*Family dysfunction* was assessed at T1 and T3 using the general functioning scale of the McMaster Family Assessment Device (FAD; Epstein *et al*., 1983). Parenting at T1 was measured using a short version of the Egna Minnen Beträffande Uppfostran (My Memories of Upbringing) for Children (EMBU-C) (Markus *et al*., 2003). The participants reported on perceived *warmth*, *overprotection* and *rejection*. As answers for both parents were highly correlated (*r* = 0.67–0.81), these were combined to mean scores.

At T3, adolescents rated parental *control* (based on Stattin and Kerr, 2000) and parental reactions to youth wrongdoing for both parents (based on Tilton-Weaver *et al*., 2010; see online Supplementary Table S1, for included items). Parental reactions consisted of the subscales *angry outbursts*, *guilt inducing* and *problem-solving* reactions. Measures of both parents were highly correlated (*r* = 0.63–0.74) and therefore combined into one mean score.

#### Peer status, affection and relationship quality (T1 and T3)

Perceived *peer status* and *affection* at T1 were assessed using the Social Production Functions Questionnaire (SPF; Ormel *et al*., 1997).

At T3, the relationship quality with peers was measured using a friendship-network interview conducted by trained researchers (based on Poulin and Pedersen, 2007). Adolescents could nominate up to seven friends, and reported on *emotional support*, *practical help* and *fights* for each friend (see online Supplementary Table S2).

#### Multidimensional functioning (T5)

Indicators for overall young adult functioning included measures assessing positive and negative functioning using existing questionnaires and questionnaires developed by TRAILS.

*Positive functioning*. *Physical health* and general *happiness* were reported in questionnaires developed by TRAILS. *Satisfaction* was assessed with a question on general life satisfaction combined with questions regarding work (Copenhagen Psychosocial Questionnaire [COPSOQ]; Kristensen *et al*., 2005) and/or romantic relationship satisfaction (Investment Model Scale [IMS]; Rusbult *et al*., 1998) if applicable. *Positive affect* was measured using the Positive and Negative Affect Schedule (PANAS) (Watson *et al*., 1988; MacKinnon *et al*., 1999). Items from the Adult Behaviour Checklist (ABCL; Achenbach and Rescorla, 2003) personal strengths subscale were used for *positive social functioning* and *personal achievement. Educational attainment* was measured with two questions on the highest diploma obtained or on the current educational level if still at school (Veldman *et al*., 2014). Finally, *daily occupation* assessed whether participants were currently working and/or studying full-time (3), part-time (2), or had no occupation (1).

*Negative functioning. Negative affect* was assessed with the PANAS (Watson *et al*., 1988; MacKinnon *et al*., 1999). The Adult Self Report (ASR) and ABCL were used to assess mental health problems (Achenbach and Rescorla, 2003). For *affective problems*, the mean scores of the depressive and anxiety problems of the Diagnostic and Statistical Manual 4th edition (DSM-IV) subscales (ASR and ABCL) were combined. Finally, the DSM-IV subscales *attention* (*deficit hyperactivity*) *problems*, *antisocial personality problems* and *avoidant personality problems* were included.

#### Covariates

Socio-economic status (*SES*) was determined by parental educational and occupational levels and family income at T1. Parental educational level was summarized in five categories. Occupational level was based on the International Standard Classification of Occupations (Ganzeboom and Treiman, 1996). Low family income was defined as a monthly net family income of less than €1135 per month, which approximately amounts to a welfare payment. Based on parental reports on their marital status, we included whether participants lived in a one-parent or two-parent household at T1. Finally, *mental health* at T1 was assessed using the mean total problems scores of the Child Behaviour Checklist (CBCL) and Youth Self Report (YSR) (Achenbach and Rescorla, 2001).

### Statistical analysis

First, confirmatory factor analysis was performed to assess the measurement model fit of young adult functioning. Two first-order latent variables were created for positive and negative functioning, which together formed the second-order variable of overall multidimensional functioning at T5. In order to accomplish model identification with two indicators of multidimensional functioning, the factor loadings for positive and negative functioning were constrained to be equal in strength, after reversing the scores for negative functioning, such that a higher score indicated less negative functioning. We allowed for correlated residuals between observed variables measured by the same instrument.

**Fig. 1.**
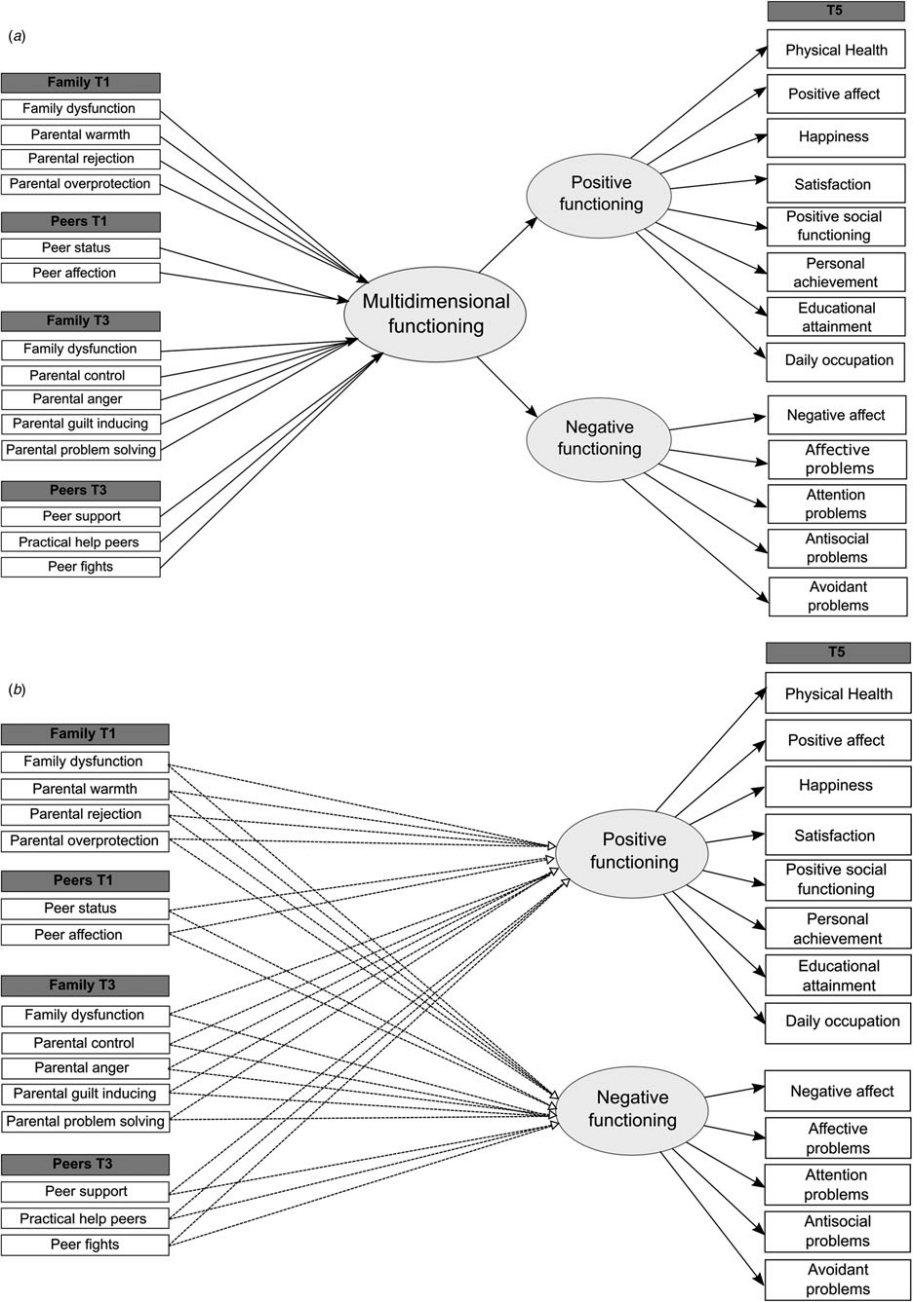
Schematic overview of the structural equation models assessing effects of the social environment on the second-order latent variable multidimensional functioning (*a*) and the two first-order latent variables positive and negative functioning (*b*). For ease of interpretation, cross-sectional and longitudinal interaction effects between family and peer environments were omitted.

Next, structural equation models (SEM) were used to examine which social experiences during adolescence predicted later functioning. Measures of the social environment (i.e. general family functioning, perceived parenting, peer status and peer relationship quality) at ages 11 (T1) and 16 (T3) were included as potential predictors of functioning at age 22 (T5). In addition to main effects, the model included cross-sectional and longitudinal interactions between the family and peer environments to investigate potential exacerbating or attenuating effects on later functioning. Note that ideally the same measures of family and peer experiences should be included for comparing T1 and T3 effects. In our study, this was only possible for family functioning. Separate analyses were carried out for overall multidimensional functioning (model 1; Fig. 1*a*) and for positive and negative functioning (model 2; Fig. 1*b*) in order to compare whether effects found in overall functioning were also present for positive or negative functioning. More parsimonious models were derived with manual backward selection by excluding the least significant main or interaction effect considering all coefficients until reaching *p* < 0.05 for all included effects (with exception of non-significant main effects corresponding to significant interaction effects).

Model estimation was based on maximum likelihood estimation with robust standard errors (MLR) in Mplus version 7.31 (Muthén and Muthén, 2015). MLR is capable of handling missing data as well as skewed distributions of outcome variables, issues both present in our data. MLR only excludes participants with missing data on all variables (*n* = 2), thus the sample size for the final models was *n* = 2228. Model fit was examined by the following criteria: the root mean square error of approximation (RMSEA), the comparative fit index (CFI) and the standardized root mean residual (SRMR). A fit is considered acceptable to good when the model achieves >0.90 on the CFI, <0.10 on RMSEA and <0.09 on SRMR (Hu and Bentler, 1999).

Considering the large number of tests performed, we applied a correction for multiple testing based on the effective number of independent tests (*M*_eff_) (Li and Ji, 2005). The *M*_eff_ was calculated separately for the number of outcome and predictor variables present in the full models using the eigenvalues of the corresponding correlation matrices (see online Supplementary Tables S3 and S4 for more information). This resulted in a total number of 75 tests (1.47 × 51 = 74.97) and a corresponding *p* value threshold of 0.05/75 = 0.00066. To avoid being overly conservative, we discuss both robustly (i.e. those effects surviving multiple testing correction: *p* < 0.00066) and nominally (*p* < 0.05) significant results. The latter should be interpreted with particular caution as replication is necessary. Significant interaction effects were probed using the Johnson–Neyman procedure (Johnson and Fay, 1950). This procedure allows us to model interactions between two continuous variables using regions of significance, thereby showing at which levels of a variable the effect of the other variable is significant.

#### Sensitivity analyses

Differences in sex, SES and family structure have been associated with both social environments and functioning (e.g. Rose and Rudolph, 2006; Bramlett and Blumberg, 2007; Almquist *et al*., 2010), and may therefore act as potential confounders. In addition, the association between social factors and functioning can be bidirectional, that is, early mental health problems may give rise to problems with family and peers as well (Meeus, 2016). Therefore, following our main analyses, we tested whether robustly and nominally significant effects remained present while controlling for sex, SES, number of parents and mental health at age 11, and tested for potential sex moderation effects.

## Results

Bivariate correlations and a graphical overview of the final SEM models including factor scores for multidimensional and positive or negative functioning are presented in the online Supplementary material (Table S5 and Figs S1 and S2). The confirmatory factor analysis of functioning measures showed that the measurement model provided a good fit to the data (χ^2^ 401.91; RMSEA 0.07; CFI 0.94; SRMR 0.06). All factor loadings were significant (*p* < 0.001) and ranged between 0.19 and 0.76 for positive functioning and 0.53 and 0.90 for negative functioning. Factor loadings for multidimensional functioning were estimated as 0.91 for both positive and negative functioning.

### Multidimensional functioning

**Fig. 2.**
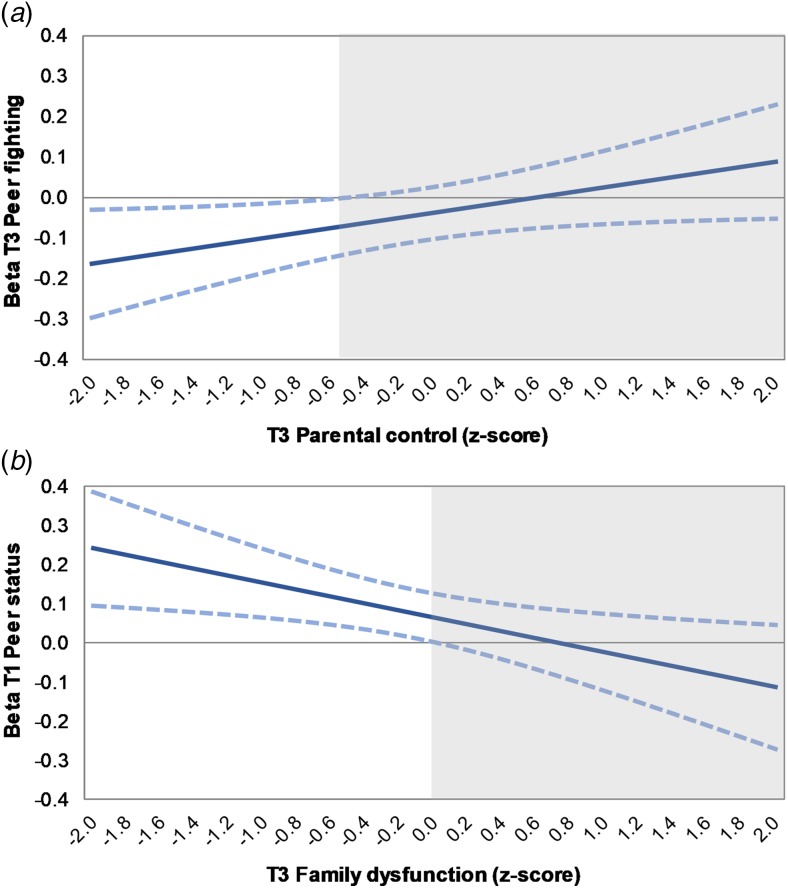
Johnson–Neyman plots showing (*a*) the conditional effect of T3 peer fighting for T3 parental control and (*b*) of T1 peer status for T3 family dysfunction on young adult multidimensional functioning. The non-shaded area indicates regions-of-significance.

Table 2 shows the standardized regression coefficients of the final SEM models. Together, adolescent family and peer experiences explained 12–15% of the variance in functioning at age 22. Multidimensional functioning was robustly predicted by T1 parental overprotection and T3 parental anger. Nominally significant associations were found with T1 family dysfunction, T1 parental warmth and T3 parental guilt inducing. Two interaction effects were found in which peer effects depended on family experiences. First, the effect of T3 peer fighting depended on T3 parental control; only when parental control was low did peer fighting significantly predict worse functioning (*z*-score <−0.06). This negative effect became stronger as parental control decreased (see Fig. 2*a*). Second, the positive effect of T1 peer status depended on T3 family dysfunction, and was only significant when family dysfunction was low (Fig. 2*b*). Here, peer status was a stronger predictor for later functioning when family dysfunction decreased. Note that these interactions were nominally significant; none survived correction for multiple testing.

**Table 2.** Results of structural equation models predicting young adult functioning

	Multidimensional functioning			Positive functioning			Negative functioning		
	*β*	95% CI	*p*	*β*	95% CI	*p*	*β*	95% CI	*p*
Family environment T1									
Family dysfunction	−0.07	(−0.12 to −0.02)	0.023	−0.09	(−0.15 to −0.04)	0.008	0.06	(0.01 to 0.11)	0.044
Parental warmth	0.06	(0.01 to 0.10)	0.043	0.08	(0.03 to 0.14)	0.013	**–**	**–**	**–**
Parental rejection	**–**	–	**–**	0.02	(−0.04 to 0.08)	0.611	**–**	**–**	**–**
Parental overprotection	**−0.13**	(**−0.18 to −0.08)**	**<0.001**	**−0.13**	(**−0.19 to −0.07)**	**<0.001**	**0.11**	**(0.07 to 0.16)**	**<0.001**
Peer environment T1									
Peer status	0.06	(0.01 to 0.11)	0.040	0.06	(0.01 to 0.12)	0.074	−0.06	(−0.11 to −0.01)	0.033
Peer affection	**–**	**–**	**–**	**–**	**–**	**–**	0.01	(−0.03 to 0.05)	0.641
Family environment T3									
Family dysfunction	**−0.14**	(**−0.20 to −0.08)**	**<0.001**	−0.09	(−0.16 to −0.02)	0.024	**0.15**	**(0.09 to 0.20)**	**<0.001**
Parental control	0.01	(−0.05 to 0.06)	0.838	–	–	–	0.05	(0.01 to 0.09)	0.063
Parental anger	**−0.13**	(**−0.18 to −0.08)**	**<0.001**	–	–	–	**0.14**	**(0.09 to 0.18)**	**<0.001**
Parental guilt inducing	−0.13	(−0.19 to −0.07)	0.001	−0.12	(−0.18 to −0.06)	0.002	**0.13**	**(0.07 to 0.19)**	**<0.001**
Parental problem solving	**–**	**–**	**–**	0.08	(0.04 to 0.13)	0.004	**–**	**–**	**–**
Peer environment T3									
Peer support	**–**	**–**	**–**	**–**	**–**	**–**	**–**	**–**	**–**
Practical help peers	**–**	**–**	**–**	**–**	**–**	**–**	**–**	**–**	**–**
Peer fights	−0.04	(−0.09 to 0.02)	0.245	−0.05	(−0.11 to 0.01)	0.137	0.03	(0.02 to 0.11)	0.246
Interaction effects									
T1 family dysfunction × T1 peer status	**–**	**–**	**–**	−0.07	(−0.11 to −0.02)	0.031	**–**	**–**	**–**
T1 parental rejection × T1 peer status	**–**	**–**	**–**	−0.07	(−0.11 to −0.02)	0.024	**–**	**–**	**–**
T1 parental overprotection × T1 peer affection	**–**	**–**	**–**	**–**	**–**	**–**	−0.06	(−0.10 to −0.02)	0.007
T3 parental control × T3 peer fights	0.06	(0.01 to 0.10)	0.041	**–**	**–**	**–**	−0.06	(−0.09 to −0.03)	0.005
T3 family dysfunction × T1 peer status	−0.09	(−0.15 to −0.03)	0.017	**–**	**–**	**–**	0.09	(0.04 to 0.14)	0.002
T1 parental rejection × T3 peer fights	**–**	**–**	**–**	−0.07	(−0.12 to −0.02)	0.024	**–**	**–**	**–**
Model fit indices									
χ^2^	862.75			960.09			960.09		
RMSEA	0.04			0.04			0.04		
CFI	0.90			0.90			0.90		
SRMR	0.05			0.04			0.04		
*R*^2^	0.15			0.12			0.14		

Structural equation models are based on maximum likelihood with robust standard error estimation (MLR), sample size *n* = 2228. For ease of interpretation, scores for negative functioning were reversed back such that a higher score indicates more negative functioning. Significant effects surviving the correction for multiple testing (*p* < 0.00066) are shown in bold.

### Positive and negative functioning

Family and peer effects on positive and negative functioning showed both general and specific effects. The effect of T3 parental guilt inducing was found on both positive and negative functioning. The direct effects of T1 parental warmth, T1 parental overprotection and T3 family dysfunction appeared to be specific for positive functioning, whereas the effects of T1 family dysfunction and T3 parental anger were found for negative functioning only. In addition, positive functioning was nominally significantly predicted by parental problem solving, which did not have an effect on either multidimensional or negative functioning.

**Fig. 3.**
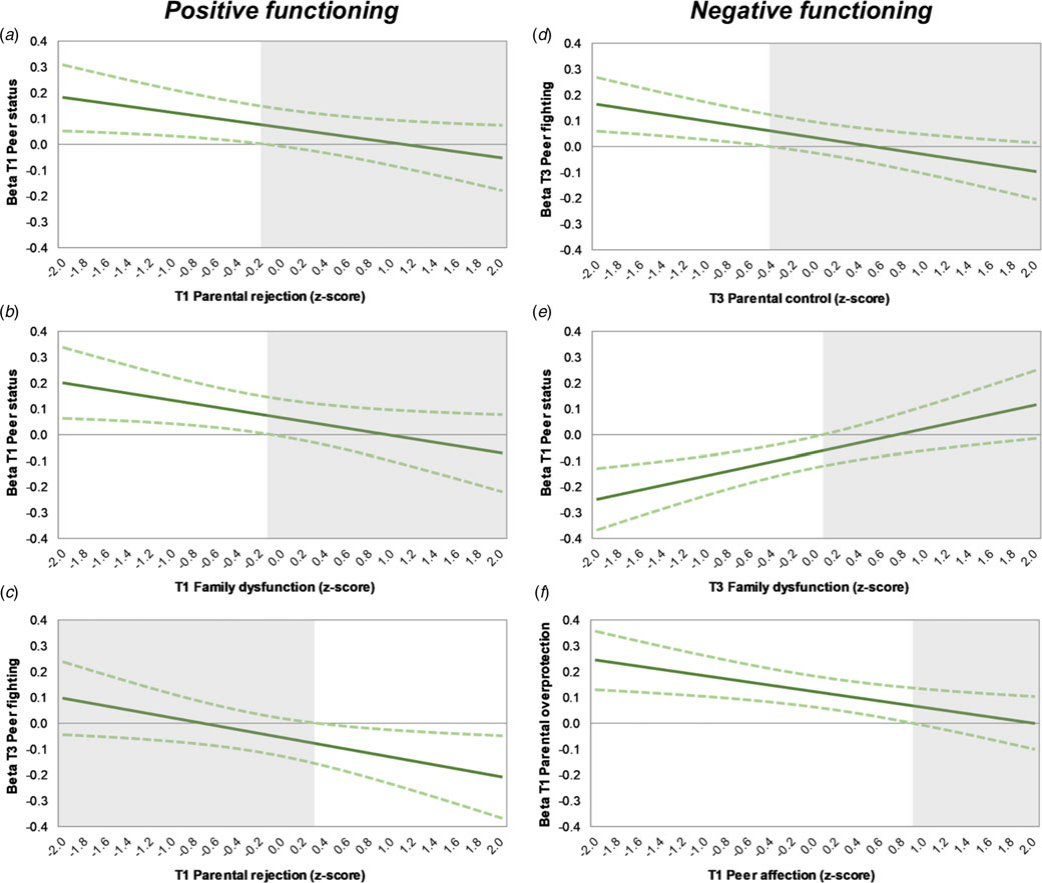
Johnson–Neyman plots showing (*a*) the conditional effect of T1 peer status for T1 parental rejection, (*b*) of T1 peer status for T1 family dysfunction, and (*c*) of T3 peer fighting for T1 parental rejection on young adult positive functioning, and (*d*) the conditional effect of T3 peer fighting for T3 parental control, (*e*) of T1 peer status for T3 family dysfunction, and (*f*) of T1 parental overprotection for T1 peer affection on young adult negative functioning (*c*). The non-shaded areas indicate regions-of-significance. For ease of interpretation, scores for negative functioning were reversed back such that a higher score indicates more negative functioning.

Associations were also found with T1 family dysfunction for positive functioning, and T1 overprotection and T3 family dysfunction for negative functioning, however these interacted with peer experiences. For negative functioning, two interactions similar to those found for multidimensional function were present (Figs 3*d* and 3*e*). In addition, negative functioning was predicted by an interaction effect between T1 peer affection and T1 parental overprotection; the negative effect of parental overprotection attenuated as peer affection increased and was no longer significant at high levels of peer affection (Fig. 3*f*). For positive functioning, three interactions were significant. Peer status predicted functioning only when T1 parental rejection or T1 family dysfunction was low (Figs 3*a* and 3*b*); and T3 peer fighting predicted poor functioning only when parental rejection was high (Fig. 3*c*). Here too interactions were found to be nominally significant at the most.

#### Sensitivity analyses

Most effects remained equal in strength, as well as statistically significant when controlling for sex, SES or number of parents in the final models, although a few dropped to non-significance (see Supplementary Tables S6–S8). In contrast, controlling for T1 mental health problems generally reduced the number of significant effects. Nearly all T1 effects became non-significant, with the exception of parental warmth for positive functioning. Additionally, for T3, the effects of family dysfunction on positive functioning and the interaction family dysfunction × T1 peer status on multidimensional functioning became non-significant (*p* > 0.07). Similar results were found when simultaneously controlling for all potential confounders. Finally, no sex moderation effects survived the correction for multiple testing.

## Discussion

This study examined to which extent family and peer experiences during adolescence jointly predict wellbeing and functioning in young adulthood. The results showed that 12–15% of the variance in functioning at age 22 was explained by adolescent family and peer experiences, of which family relations during early and mid-adolescence (ages 11 and 16) were most predictive. Young adolescents reporting less negative family experiences overall demonstrated better adjustment in young adulthood. Peer experiences did not independently predict functioning. Furthermore, certain family and peer experiences appeared interdependent in their prediction of later functioning.

Parental overprotection and negative parental reactions robustly predicted future functioning, which is in line with previous research (e.g. Huppert *et al*., 2010; Tilton-Weaver *et al*., 2010; Baker and Hoerger, 2012). Parental overprotection is thought to limit a child's opportunity to independently explore the world and to develop healthy self-regulating and coping strategies (Thomasgard and Metz, 1993). Similarly, negative parental reactions to misbehaviour, such as anger, are thought to increase feelings of being restricted and decrease feelings of connectedness (Tilton-Weaver *et al*., 2010). Our findings are in agreement with the McMaster model of family functioning, which considers affective responsiveness and involvement as core functions of families; with inappropriate responses and involvement viewed as detrimental for a child's development (Epstein *et al*., 1978).

In line with ecological and social systems theories, we found that the effects of peer contexts can depend on the family context and *vice versa* (Bronfenbrenner, 1979; Hartup, 1989). For example, the negative effect of peer fighting was absent in case of high parental control or low parental rejection. In addition, the negative effect of parental overprotection was no longer significant at high levels of peer affection. This suggests both family and peer contexts may function as ‘buffers’, in line with previous studies on adolescent functioning (Gauze *et al*., 1996; Lansford *et al*., 2003; Gaertner *et al*., 2010; Sentse *et al*., 2010; Véronneau and Dishion, 2010; Trudeau *et al*., 2012). According to the stress-buffering model, supportive social contexts – such as positive parenting characterized by low levels of rejection – can protect against the adverse effects of stressful events by attenuating the appraisal of stress. That is, the observation that others are willing to invest in a person may help to decrease the experience of stress (Cohen and Wills, 1985). Note that these effects can also be interpreted as reflecting a dual-hit pattern, in which negative family or peer effects are particularly relevant when experiences in the other context are negative as well. This is line with the idea that the same variable can act both as buffer and risk factor, depending on what ‘side of the coin’ is focused on (Lösel and Farrington, 2012).

Finally, we found that the positive effect of peer status was only present in the absence of negative family experiences, including low parental rejection and family dysfunction. As such the positive effect of peer status does not appear to be able to compete with negative family experiences. Together these findings suggest that family and peer experiences can interact in different ways. Risk factors in one context can be exacerbated by negative experiences or buffered by positive experiences in the other context, and protective factors in one context can be overshadowed by negative experiences in the other context. Furthermore, interactions between parent and peer contexts appear to be circumscribed, that is, holding for some aspects of family and peer experiences, but not for others. As none of the interaction effects survived the correction for multiple testing, future studies are necessary to investigate the robustness of these findings.

When comparing the relative contribution of family and peers on future functioning, we primarily found family effects. That is, when taking both contexts into account, the type of peer relations we measured at ages 11 and 16 did not seem incremental to the predictive ability of family experiences for young adult functioning. As such, our findings suggest that adolescent family experiences may be more important for future functioning than adolescent peer relations. So far, little research has considered adolescent family and peer relations in one model when predicting (young) adult adjustment. Moreover, studies that have done so often included other aspects of social relations than studied here, such as deviant peer behaviours or romantic relations, or focused specifically on substance abuse as outcome, making it difficult to compare results. For example, in line with our findings, two studies reported stronger family than peer effects for positive social relations and autonomy support (der Giessen *et al*., 2014; Jones *et al*., 2016). Seemingly inconsistent with our study is that Jones *et al*. (2016) reported particularly strong effects of peer drug abuse on future functioning. This suggests that the relative contributions of family and peer relations on future adjustment are likely to depend on the type of peer factors (e.g. peer relationship quality *v.* deviant peer behaviours).

In addition to the kind of peer factors involved, the relative importance of family and peer relations may also depend on developmental timing. Whereas some have argued that adolescence marks a shift from parents to peers as the primary source of support (Furman and Buhrmester, 1992), others have emphasized the continued importance of the family environment (Raja *et al*., 1992). In agreement with the latter, our findings stress the role of family experiences at both ages 11 and 16. Although the use of different measures over time prevents us from drawing firm conclusions. Possibly, the importance of peer relationship quality for adult functioning has shifted from adolescence towards young adulthood. In recent decades, the period of adolescence has lengthened, with a protracted transition into adulthood in terms of leaving the parental home and becoming financially independent (Steinberg, 2014). It is plausible that peers matter most in this transition to independence and beyond. Indeed, studies have shown that the effects of adolescent peer relations are mediated by current (romantic) peer relations in young adulthood (Englund *et al*., 2011; Jones *et al*., 2016). Overall, considering the scarcity of studies comparing the synergy of long-term family and peer effects, further research, also beyond the traditional end of adolescence, is warranted.

We looked whether the effects found for overall multidimensional functioning were also present for positive or negative functioning. Most effects appeared similar for both sides of functioning. However, effects of parental warmth and parental problem solving – the only two positively framed family experiences – seemed to be specific for positive functioning, while parental anger specifically predicted negative functioning. This might suggest that positive social experiences may be especially relevant for predicting young adult positive adjustment, while negative experiences are more relevant for negative functioning. Such a pattern of specificity is not unlikely considering that positively framed experiences and behaviours usually tend to be skewed to (i.e. contain more variance in) the positive side of the distribution, and negatively framed variables to the negative side. That said, positive functioning was also predicted by negative social experiences, thus negative experiences and behaviours do not merely predict negative outcomes. In all, our findings are in line with the idea that mental health problems and wellbeing can have both overlapping and distinctive correlates (e.g. Kinderman *et al*., 2015; Patalay and Fitzsimons, 2016), and suggests that targeting predictors of one domain shall not necessarily benefit the other.

### Strengths and limitations

This study should be viewed in light of certain strengths and limitations. Strong points include the longitudinal prospective study design and multidimensional approach encompassing positive and negative functioning. Moreover, we extended previous literature by accounting for both family and peer factors, which were measured at two developmentally relevant time points in adolescence. Some limitations should be noted as well. First, although we included multiple informants when possible, most data were based on self-reported questionnaires. Observational measures of social experiences and adult functioning would have been a valuable addition. Nevertheless, it can be argued that the way a person perceives social relations is likely to be more important for one's development compared with actual, but unperceived social experiences (e.g. Laceulle *et al*., 2015). Second, except for family dysfunction, different measures of the social environment were used in TRAILS at each time point. Consequently, we were unable to investigate whether specific aspects of social experiences, such as parental overprotection or peer popularity, have the same influence in early and mid-adolescence. Third, several factors may mediate the associations found between adolescent social experiences and later functioning which have not been investigated in the current study. Future research is needed to investigate such mediating factors in more detail.

Another issue that should be pointed out is that while our longitudinal design allows for temporal ordering, we cannot infer causality from the reported findings. It is certainly plausible that the adolescent social experiences studied here are a direct cause of later maladjustment; however, they can also be markers of true underlying causal factors of later functioning, such as genetic predisposition or prior maladjustment. To account for potential reverse causality, we performed sensitivity analyses with baseline mental health problems included in the model. Although correcting for functioning before age 11 would have been preferable, such measures were not available within our sample. The results showed that most effects during mid-adolescence were not the result of initial levels of functioning. However, most social experiences in early adolescence and the interplay between parental control and peer fighting no longer predicted future functioning. As such, here we cannot rule out the possibility that mental health at age 11 may have caused problems in adolescent family and peer relations. The sensitivity analyses further showed that overall differences in sex, SES or number of parents do not explain the found effects either. Further longitudinal research including multiple assessments of functioning over time is necessary to elucidate the potential mechanisms that play a role in the link between early social experiences and later functioning.

To conclude, the results indicate that both family and peer relations during adolescence can be predictive for young adult functioning, but that especially adolescent family experiences, such as parental overprotection and family dysfunction, are associated with young adult functioning. The findings further suggest that certain family and peer experiences are interdependent in their prediction of later functioning, which highlights the importance of considering the relative effects of one context in relation to the other. Nevertheless, despite a comprehensive assessment, adolescent family and peer experiences played a modest role in predicting young adult functioning. Thus, having negative family or peer experiences during adolescence does not necessarily mean one will function worse in young adulthood. At least, for adolescents growing up in fairly normal circumstances such as the participants included in this study from the TRAILS population cohort. That is, more extreme social experiences, especially at the adverse end, may have a greater predictive value for later functioning than the effects found here suggest. Future studies are necessary to further investigate whether the relative contribution and interdependence of families and peers holds true for other social experiences as well.

## Supplementary material

For supplementary material accompanying this paper visit https://doi.org/10.1017/S0033291718001976. click here to view supplementary material
