# Supplementary material for: Beyond not bad or just okay: social predictors of young adults’ wellbeing and functioning (a TRAILS study)
Source: Psychol Med. 2018 Sep 19;49(9):1459–69. doi: 10.1017/S0033291718001976 (PMC6541871; doi:10.1017/S0033291718001976)
Supplement: Supplementary file 1 [file S0033291718001976sup001.docx]

***Online Supplemental Material***

**Beyond not bad or just okay: Social predictors of young adults’ wellbeing and functioning. A TRAILS study**

Richards, J.S. ^*^, Hartman, C.A., Jeronimus, B.F., Ormel, J. , Reijneveld, S.A. , Veenstra, R., Verhulst, F.C., Vollebergh, W.A.M., Oldehinkel, A.J.

**Content**

1. Measures…………………………………………………………………………..2

Table S1. Assessment parenting T3………………………………………………3

Table S2. Assessment peer relationship quality T3……………………………....4

1. Multiple testing correction: the *M_eff_* procedure…………………………………...7

Table S3. Eigenvalues for outcome variables……………………………….........8

Table S4. Eigenvalues for predictor variables……………………………….........9

Table S5. Bivariate correlations between study variables………………….........11

1. Graphical overview final SEM models………………………………………….13
2. Sensitivity analyses……………………………………………………………...15

Table S6. Results of structural equation models predicting young adult multidimensional functioning, controlling for sex, SES, number of parents,
and mental health at age 11……………………………………………………...16

Table S7. Results of structural equation models predicting young adult
positive functioning, controlling for sex, SES, number of parents,
and mental health at age 11……………………………………………………...17

Table S8. Results of structural equation models predicting young adult
negative functioning, controlling for sex, SES, number of parents,
and mental health at age 11…………………….……………………………….18

1. References………………………………………………………………………19

**1. Measures**

**Family functioning and parenting (T1 and T3).** *Family dysfunction* was assessed at T1 and T3 using the general functioning scale of the McMaster Family Assessment Device (FAD; Epstein *et al.* 1983). This scale assesses emotional relationships and functioning within the family, based on the six dimensions of the McMaster model: problem solving, communication, roles, affective responsiveness, affective involvement, and behavioural control (Epstein *et al.* 1983). Parenting at T1 was measured using a short version of the Egna Minnen Beträffande Uppfostran (My Memories of Upbringing) for Children (EMBU-C) (Markus *et al.* 2003). The participants reported on perceived *warmth*, *overprotection*, and *rejection.* Emotional warmth is defined by affection, attention, and support. Rejection is characterized by hostility, punishment, derogation, and blaming of subject. Overprotection is characterized by fearfulness and anxiety for the child’s safety, guilt engendering, and intrusiveness. As answers for both parents were highly correlated (*r* = 0.67 - 0.81), these were combined to mean scores. The validity of the EMBU-C has been reported in Markus et al. (2003).

At T3, adolescents rated parental control (based on Stattin & Kerr 2000) and parental responses to youth wrongdoing for both parents (based on Tilton-Weaver *et al.* 2010). Exact items included in TRAILS for the assessment of parental control and reactions at T3 are described in Table S1 Parental *control* captured the information required by parents regarding adolescents’ whereabouts, companions, and activities. High test-retest reliability has been reported for the original parental control scale (Stattin & Kerr 2000). Parental reactions consisted of the subscales *angry outbursts*, *guilt inducing,* and *problem-solving* reactions. Measures of both parents were highly correlated (*r* = 0.63 - 0.74) and therefore combined into one mean score. Concurrent and predictive validity has been found for the original parental reactions scale (Tilton-Weaver *et al.* 2010).

**Peer status, affection and relationship quality (T1 and T3).** Perceived *peer status* and *affection* at T1 were assessed using the Social Production Functions Questionnaire (SPF; Ormel *et al.* 1997). Perceived affection was based on two subscales of the SPF: affection and behavioural confirmation, which were highly correlated (*r* = .77), and therefore combined into one mean affection score. A longer version of the SPF has been extensively validated (Nieboer *et al.* 2005).

At T3 the relationship quality with peers was measured using a friendship-network interview conducted by trained researchers (based on Poulin & Pedersen 2007). Adolescents could nominate up to seven friends, and reported on emotional *support*, *practical help*, and *fights* for each friend. Exact items included in TRAILS for the assessment of peer relationship quality at T3 are described in Table S2. Mean scores were calculated over all nominated friends.

**Table S1.** Items included measuring parental control and reactions T3

| *Construct* | *Items* |
| --- | --- |
| Parental control | Must you ask your parents before you can make plans with friends about what you will do on a Saturday night? |
|  | If you have been out past curfew, do your parents require that you explain why and tell who you were with? |
|  | Do you need to have your parents' permission to stay out late on a weekday evening? |
|  | Do your parents demand that they know where you are in the evenings, who you are going to be with, and what you are going to do? |
|  | If you go out on a Saturday evening, must you inform your parents beforehand about who will be along as well as where you will be going? |
| Parental reactions: |  |
| Angry outbursts | Your father/ mother has outbursts of anger and tells you off |
|  | Your father/ mother has a hard time controlling his/her irritation |
|  | Your father/ mother quarrels and complains loudly |
| Guilt inducing | Your father/ mother avoids you |
|  | Your father/ mother is silent and cold toward you |
|  | Your father/ mother doesn’t talk to you until after a long while |
| Problem solving | Your father/ mother is clear about what she [he] thinks, but is open to discussions |
|  | Your father/ mother honestly wants to understand why you did what you did |
|  | Your father/ mother tries to understand how you thought and felt |
|  | Your father/ mother tries to talk through it without creating new conflicts |

**Table S2.** Items included measuring parental control and reactions T3

| *Construct* | *Items* |
| --- | --- |
| Peer support | Does [name] help you when you are feeling down? |
| Practical help peers | Does [name] help you practically? |
| Peer fights | Do you and [name] have fights? |

**Multidimensional functioning T5**. Indicators for overall young adult functioning included measures assessing positive and negative functioning using existing questionnaires and questionnaires developed by TRAILS.

***Positive functioning.*** Measures of physical health, subjective wellbeing (happiness, satisfaction, positive affect) and socio-academic functioning (positive social functioning, personal achievement, educational attainment, daily occupation) were used as indicators of positive functioning. Participants were asked to report their current *physical health* (last 30 days) on a 4-point scale (bad – good) and general *happiness* on a 10-point scale (very unhappy – very happy). *Satisfaction* was assessed by a question on general life satisfaction (10-point scale: very unsatisfied – very satisfied), combined with questions regarding work and/ or romantic relationship satisfaction if applicable. For work satisfaction 3 items were selected from the Copenhagen Psychosocial Questionnaire (COPSOQ; Kristensen *et al.* 2005) : “Is your work meaningful?”, “Do you feel that the work that you do is important?” (4 point-scale: to a very small extent – to a very large extent), and “How pleased are you with your job as a whole, everything taken into consideration?” (3-point scale: very unsatisfied – very satisfied). Good validity and reliability of the COPSOQ has been reported by Kristensen and colleagues (2005). For romantic relationship satisfaction two items were selected from the Investment Model Scale (IMS; Rusbult *et al.* 1998): “I am satisfied with my relationship”, and “My relationship gives me what I need with respect to intimacy, friendship, etc.” (7-point scale: strongly disagree – strongly agree). The IMS has been shown to be a valid and reliable instrument (Rusbult *et al.* 1998). Each satisfaction domain (life, work, relationship) was given equal weight by first recoding all items to reflect the same scale, then calculating mean scores per domain, and subsequently combining all mean scores into an overall mean satisfaction score. This overall satisfaction score correlated highly with each item (*r* = .51 - 74). For 578 participants their overall satisfaction score was based on all satisfaction domains, for 705 participants on two domains (*n =* 242 based on life satisfaction and romantic relationship, *n* = 461 based on life and work satisfaction, *n* = 2 based on work and romantic relationship), and for 230 participants on one domain (*n* = 216 based on life satisfaction only, *n* = 2 based on romantic relationship only, *n* = 12 based on work satisfaction only). The Positive and Negative Affect Schedule (PANAS) (Watson *et al.* 1988; MacKinnon *et al.* 1999) was used to measure *positive* *affect.* Participants indicated the extent to which they had experienced positive (e.g. interested) and negative feelings (e.g. distressed) during the last month (5-point scale; never – very often). Adequate validity and reliability has been reported for the PANAS (Crawford & Henry 2004). Items from the Adult Behaviour Checklist (ABCL; Achenbach & Rescorla 2003) personal strengths subscale were used for *positive social functioning* (i.e., “Meets responsibilities to his/her family”, “Enjoys being with people”, “Likes to help others”, “Tries to be fair to others”) and *personal achievement* (i.e. “Likes to try new things”, “Makes good decisions”, “Makes good use of his/her opportunities”, “Works up to ability”, “Can do certain things better than other people”). All items were scored on a 3-point scale (not true – very or often true) referring to the past 6 months. Adequate reliability and validity has been reported for the ABCL (Achenbach & Rescorla 2003). *Educational attainment* was measured with two questions on the highest diploma obtained or on the current educational level if still at school. Educational attainment was categorized into primary (1), lower secondary (2), higher secondary (3), higher vocational (tertiary) (4), and university (5) (Veldman *et al.* 2014). Finally, *daily occupation* assessed whether participants were currently working and/or studying full-time (3), part-time (2), or had no occupation (1). Participants reported whether they were currently in school or not, if so studying full-time or part-time; whether they had a paid job in the last month and if so how many hours they worked (20 hours or more was considered full-time occupation). Participants working *and* studying part-time were rated as full-time.

***Negative functioning.*** Measures of negative affect and mental health problems (affective, attention, antisocial personality, and avoidant personality problems) were used as indicators of negative functioning. *Negative affect* was assessed with the PANAS (Watson *et al.* 1988; MacKinnon *et al.* 1999), as described above*.* The Adult Self Report (ASR) and Adult Behaviour Checklist (ABCL) were used to assess mental health problems (Achenbach & Rescorla 2003). The ASR and ABCL contain a list of behaviours and problems scored on a 3-point scale (not true to very or often true) referring to the past 6 months. ABCL and ASR scores were combined when both available, as multi-informant information provides a better prediction of mental health problems (Verhulst & Ende 1992). For *Affective problems,* the mean scores of the depressive and anxiety problems of the Diagnostic and Statistical Manual 4^th^ edition (DSM-IV) subscales (ASR and ABCL) were combined. Finally, the DSM-IV subscales *attention (deficit hyperactivity) problems*, *antisocial personality problems*, and *avoidant personality problems* were included. Like the ABCL, adequate reliability and validity has been found for the ASR (Achenbach & Rescorla 2003).

**Covariates.** Socio-economic status (*SES*) was determined by parental educational and occupational levels and family income at T1. Parental educational level was summarized in five categories. Occupational level was based on the International Standard Classification of Occupations (Ganzeboom & Treiman 1996). Low family income was defined as a monthly net family income of less than 1135 euro’s per month, which approximately amounts to a welfare payment. SES was measured as the average of the five items (*α* = .84). Based on parental reports on their marital status, we included whether participants lived in a one-parent or two-parent household at T1. Finally, *Mental health* at T1 was assessed using the mean total problems scores of the Child Behaviour Checklist (CBCL) and Youth Self Report (YSR; Achenbach & Rescorla 2001). Reliability and validity for the CBCL and YSR has been demonstrated (Achenbach & Rescorla 2001).

1. **Multiple testing correction: the *M_eff_* procedure**

Correction for multiple testing was based on adjusting tests using the effective number of independent comparisons (*M_eff_*) (Li & Ji 2005). The main idea behind the *M_eff_* procedure is that the effective number of independent comparisons is determined by means of the correlations between the tested variables, using the sum of the Eigenvalues of the correlation matrix. In the scenario that all correlations between the variables are equal to zero, the *M_eff_* adjusted *p*-value is equivalent to a Bonferroni correction. However, when all correlations are one, the adjusted *p*-value is equal to the nominal significance threshold (*p* < .05). Thus, the *M_eff_* procedure is particularly suited for correlated tests, such as correlations between multiple related behaviour or parenting measures.

We calculated the *M_eff_* separately for the number of outcome and predictor variables present in the full models. Predictor variables included both main and interaction effects. Tables S1 and S2 show the eigenvalues for the outcome and predictor variables, which were calculated using the online application offered on [www.junningli.org](http://www.junningli.org/). The *M_eff_* for the outcome variables was determined to be 1.47 and for the predictor variables 51. The total number of independent tests was then calculated by multiplying the *M_eff_* of the outcome and predictor variables: 1.47 * 51 = 74.97 leading to a corresponding *p*-value threshold of .05/75 = .00066.

**Table S3.** Eigenvalues for outcome variables

|  | *Outcome* | *Eigenvalues* |
| --- | --- | --- |
| 1 | Multidimensional functioning | 2.75 |
| 2 | Positive functioning | 0.07 |
| 3 | Negative functioning | 0.18 |

**Table S4**. Eigenvalues for predictor variables

|  | *Predictors* | *Eigenvalues* |
| --- | --- | --- |
| 1 | Family dysfunction T1 | 4.6217 |
| 2 | Parental warmth T1 | 2.9220 |
| 3 | Parental rejection T1 | 2.3650 |
| 4 | Parental overprotection T1 | 2.2025 |
| 5 | Family dysfunction T3 | 2.2004 |
| 6 | Parental control T3 | 2.0535 |
| 7 | Parental angry outbursts T3 | 1.9667 |
| 8 | Parental problem solving T3 | 1.7838 |
| 9 | Parental guilt inducing T3 | 1.7033 |
| 10 | Peer status T1 | 1.6444 |
| 11 | Peer affection T1 | 1.5539 |
| 12 | Peer support T3 | 1.4664 |
| 13 | Practical help peers T3 | 1.4074 |
| 14 | Peer fights T3 | 1.3785 |
| 15 | Family dysfunction T1 x Peer status T1 | 1.3205 |
| 16 | Warmth T1 x Peer status T1 | 1.2814 |
| 17 | Rejection T1 x Peer status T1 | 1.2146 |
| 18 | Overprotection T1 x Peer status T1 | 1.1913 |
| 19 | Family dysfunction T1 x Peer affection T1 | 1.1446 |
| 20 | Warmth T1 x Peer affection T1 | 1.1282 |
| 21 | Rejection T1 x Peer affection T1 | 1.1071 |
| 22 | Overprotection T1 x Peer affection T1 | 1.0972 |
| 23 | Family dysfunction T3 x Peer support T3 | 1.0226 |
| 24 | Control T3 x Peer support T3 | 1.0119 |
| 25 | Angry outbursts T3 x Peer support T3 | 0.9882 |
| 26 | Problem solving T3 x Peer support T3 | 0.9157 |
| 27 | Guilt inducing T3 x Peer support T3 | 0.8791 |
| 28 | Family dysfunction T3 x Peer help T3 | 0.8455 |
| 29 | Control T3 x Peer help T3 | 0.8317 |
| 30 | Angry outbursts T3 x Peer help T3 | 0.7718 |

**Table S4 – continued.** Eigenvalues for predictor variables

|  | *Predictors* | *Eigenvalues* |
| --- | --- | --- |
| 31 | Problem solving T3 x Peer help T3 | 0.7366 |
| 32 | Guilt inducing T3 x Peer help T3 | 0.6897 |
| 33 | Family dysfunction T3 x Peer fights T3 | 0.6742 |
| 34 | Control T3 x Peer fights T3 | 0.6653 |
| 35 | Angry outbursts T3 x Peer fights T3 | 0.6443 |
| 36 | Problem solving T3 x Peer fights T3 | 0.6341 |
| 37 | Guilt inducing T3 x Peer fights T3 | 0.6283 |
| 38 | Family dysfunction T1 x Peer support T3 | 0.5967 |
| 39 | Warmth T1 x Peer support T3 | 0.5715 |
| 40 | Rejection T1 x Peer support T3 | 0.5580 |
| 41 | Overprotection T1 x Peer support T3 | 0.5361 |
| 42 | Family dysfunction T1 x Peer help T3 | 0.5260 |
| 43 | Warmth T1 x Peer help T3 | 0.4988 |
| 44 | Rejection T1 x Peer help T3 | 0.4716 |
| 45 | Overprotection T1 x Peer help T3 | 0.4671 |
| 46 | Family dysfunction T1 x Peer fights T3 | 0.4374 |
| 47 | Warmth T1 x Peer fights T3 | 0.4259 |
| 48 | Rejection T1 x Peer fights T3 | 0.4028 |
| 49 | Overprotection T1 Peer fights T3 | 0.3657 |
| 50 | Family dysfunction T3 x Peer status T1 | 0.3469 |
| 51 | Control T3 x Peer status T1 | 0.3314 |
| 52 | Angry outbursts T3 x Peer status T1 | 0.3199 |
| 53 | Problem solving T3 x Peer status T1 | 0.2924 |
| 54 | Guilt inducing T3 x Peer status T1 | 0.2439 |
| 55 | Family dysfunction T3 x Peer affection T1 | 0.2402 |
| 56 | Control T3 x Peer affection T1 | 0.1945 |
| 57 | Angry outbursts T3 x Peer affection T1 | 0.1865 |
| 58 | Problem solving T3 x Peer affection T1 | 0.1756 |
| 59 | Guilt inducing T3 x Peer affection T1 | 0.1177 |

**Table S5.** Bivariate correlations between sex, young adult functioning, and family and peer experiences

|  | 1 | 2 | 3 | 4 | 5 | 6 | 7 | 8 | 9 | 10 | 11 | 12 | 13 | 14 | 15 | 16 | 17 | 18 | 19 | 20 |
| --- | --- | --- | --- | --- | --- | --- | --- | --- | --- | --- | --- | --- | --- | --- | --- | --- | --- | --- | --- | --- |
| 1. Sex^1^ | 1.00 |  |  |  |  |  |  |  |  |  |  |  |  |  |  |  |  |  |  |  |
| 2. SES T1 | -.03 | 1.00 |  |  |  |  |  |  |  |  |  |  |  |  |  |  |  |  |  |  |
| 3. Number of parents T1 | .01 | **.22** | 1.00 |  |  |  |  |  |  |  |  |  |  |  |  |  |  |  |  |  |
| 4. Mental health T1 | ***.07*** | **-.15** | **-.12** | 1.00 |  |  |  |  |  |  |  |  |  |  |  |  |  |  |  |  |
| 5. Multidimensional functioning T5 | **.20** | **.10** | **.16** | **-.49** | 1.00 |  |  |  |  |  |  |  |  |  |  |  |  |  |  |  |
| 6. Positive functioning T5 | .02 | .20 | **.15** | **-.41** | **.98** | 1.00 |  |  |  |  |  |  |  |  |  |  |  |  |  |  |
| 7. Negative functioning T5^2^ | **.31** | *.06* | **.14** | **-.43** | **.98** | **.86** | 1.00 |  |  |  |  |  |  |  |  |  |  |  |  |  |
| 8. Physical health | **.11** | .04 | .05 | **-.19** | **.52** | **.55** | **.46** | 1.00 |  |  |  |  |  |  |  |  |  |  |  |  |
| 9. Happiness | .05 | -.01 | .05 | **-.21** | **.69** | **.77** | **.59** | **.33** | 1.00 |  |  |  |  |  |  |  |  |  |  |  |
| 10. Satisfaction | ***-.09*** | -.02 | .04 | **-.17** | **.51** | **.60** | **.44** | **.26** | **.60** | 1.00 |  |  |  |  |  |  |  |  |  |  |
| 11. Positive affect | -.01 | *.06* | *.05* | **-.12** | **.41** | **.54** | **.33** | **.18** | **.38** | **.31** | 1.00 |  |  |  |  |  |  |  |  |  |
| 12. Positive social functioning | **-.12** | **.10** | .03 | **-.09** | **.22** | **.28** | **.17** | *.06* | **.13** | **.10** | **.11** | 1.00 |  |  |  |  |  |  |  |  |
| 13. Personal achievement | **-.17** | **.19** | .02 | **-.13** | **.22** | **.33** | **.15** | *.06* | **.14** | **.15** | **.17** | **.42** | 1.00 |  |  |  |  |  |  |  |
| 14. Educational attainment | -.02 | **.47** | *.06* | **-.18** | **.21** | **.28** | **.16** | **.10** | .04 | .05 | **.13** | **.14** | **.37** | 1.00 |  |  |  |  |  |  |
| 15. Daily occupation study/work | .03 | **.15** | *.06* | **-.12** | **.24** | **.28** | **.20** | **.10** | **.13** | **.12** | **.12** | **.11** | **.21** | **.38** | 1.00 |  |  |  |  |  |
| 16. Negative affect | **-.21** | .02 | *.07* | **-.20** | **.71** | **.64** | **.76** | **-.34** | **-.39** | **-.28** | **-.09** | *-.06* | -.03 | -.02 | *-.06* | 1.00 |  |  |  |  |
| 17. Affective problems | **-.23** | **.03** | **.09** | **-.31** | **.91** | **.78** | **.96** | **-.38** | **-.50** | **-.36** | **-.27** | **-.16** | **-.17** | **-.10** | **-.17** | **.59** | 1.00 |  |  |  |
| 18. Attention problems | *.06* | ***.08*** | ***.08*** | **-.36** | **.59** | **.52** | **.59** | **-.27** | **-.26** | **-.27** | **-.10** | **-.18** | **-.29** | **-.21** | **-.14** | **.38** | **.54** | 1.00 |  |  |
| 19. Antisocial personality problems | **.13** | **.13** | *.05* | **-.32** | **.53** | **.49** | **.51** | **-.23** | **-.27** | **-.24** | **-.09** | **-.23** | **-.32** | **-.30** | **-.14** | **.31** | **.43** | **.63** | 1.00 |  |
| 20. Avoidant personality problems | **-.12** | .04 | *.06* | **-.27** | **.71** | **.63** | **.72** | **-.28** | **-.44** | **-.33** | **-.29** | **-.23** | **-.18** | **-.09** | **-.16** | **.42** | **.67** | **.38** | **.33** | 1.00 |
| 21. Family dysfunction T1 | .03 | **-.20** | **-.15** | **.25** | **-.20** | **-.21** | **-.16** | -.03 | ***-.08*** | -.02 | **-.10** | **-.11** | **-.13** | **-.14** | -.05 | ***.09*** | **.13** | **.15** | **.18** | **.10** |
| 22. Parental warmth T1 | **-.10** | **.15** | ***.06*** | **-.23** | **.11** | **.20** | ***.06*** | *.06* | *.06* | .05 | **.18** | ***.07*** | **.11** | **.16** | .03 | -.01 | -.03 | ***-.07*** | **-.10** | **-.09** |
| 23. Parental rejection T1 | **.11** | -.03 | .04 | **.45** | **-.21** | **-.19** | **-.18** | ***-.08*** | **-.10** | ***-.08*** | *-.06* | ***-.07*** | **-.11** | ***-.08*** | -.01 | ***.08*** | **.11** | **.18** | **.23** | **.09** |
| 24. Parental overprotection T1 | *.05* | **-.09** | .03 | **.30** | **-.19** | **-.16** | **-.16** | **-.12** | ***-.08*** | ***-.07*** | .01 | -.05 | ***-.09*** | -.04 | -.03 | **.12** | **.11** | **.15** | **.16** | ***.07*** |
| 25. Peer status T1 | ***-.07*** | -.01 | .00 | **-.09** | *.05* | *.05* | *.04* | .03 | *.06* | .03 | .04 | .02 | .03 | -.04 | -.02 | .03 | ***-.08*** | *-.05* | -.01 | **-.10** |
| 26. Peer affection T1 | **-.18** | .02 | .01 | **-.23** | *.05* | **.10** | .03 | .02 | *.06* | .05 | **.11** | .04 | .02 | .02 | -.02 | .01 | .00 | *-.05* | ***-.07*** | *-.06* |
| 27. Family dysfunction T3 | -.02 | **-.13** | -.04 | **.19** | **-.24** | **-.18** | **-.21** | -.01 | **-.11** | -.06 | *-.06* | **-.15** | **-.11** | **-.11** | -.01 | ***.10*** | **.17** | **.18** | **.22** | **.14** |
| 28. Parental control T3 | **-.24** | **.15** | **.12** | *-.05* | -.02 | **.11** | *-.06* | .02 | .03 | *.06* | **.10** | *.07* | **.10** | **.17** | .05 | *.07* | ***.07*** | -.03 | **-.10** | .01 |
| 29. Parental anger T3 | **-.17** | .03 | .02 | **.14** | **-.26** | **-.12** | **-.26** | **-.16** | **-.10** | *-.06* | *.06* | -.05 | .03 | .05 | -.01 | **.22** | **.22** | **.20** | **.17** | **.12** |
| 30. Parental guilt inducing T3 | -.02 | -.02 | ***-.08*** | **.11** | **-.27** | **-.22** | **-.24** | ***-.08*** | **-.14** | ***-.08*** | *-.06* | ***-.08*** | -.05 | -.05 | -.02 | **.13** | **.19** | **.18** | **.19** | **.13** |
| 31. Parental problem solving T3 | **-.09** | **.18** | *.06* | **-.12** | **.11** | **.21** | *.06* | ***.09*** | ***.08*** | .03 | **.15** | ***.07*** | *.07* | **.17** | *.06* | *-.06* | -.01 | ***-.08*** | **-.15** | -.02 |
| 32. Peer support T3 | **-.36** | -.05 | -.04 | -.03 | .02 | ***.07*** | .00 | .00 | .03 | .06 | .05 | **.10** | *.07* | -.04 | -.01 | .05 | .03 | .00 | .00 | *-.06* |
| 33. Practical help peers T3 | **-.17** | -.02 | .01 | -.03 | .05 | **.09** | .03 | .03 | .05 | .02 | *.06* | .03 | .01 | .02 | .01 | -.01 | -.02 | -.01 | -.05 | *-.06* |
| 34. Peer fights T3 | **-.14** | *-.06* | .03 | **.12** | **-.13** | **-.13** | **-.12** | **-.14** | -.05 | .00 | .03 | -.03 | *-.07* | **-.10** | -.02 | **.16** | ***.09*** | ***.09*** | **.11** | .04 |

**Table S5 – continued.** Pairwise correlations between sex, young adult functioning, and family and peer experiences

|  | 21 | 22 | 23 | 24 | 25 | 26 | 27 | 28 | 29 | 30 | 31 | 32 | 33 | 34 |
| --- | --- | --- | --- | --- | --- | --- | --- | --- | --- | --- | --- | --- | --- | --- |
| 21. Family dysfunction T1 | 1.00 |  |  |  |  |  |  |  |  |  |  |  |  |  |
| 22. Parental warmth T1 | **-.16** | 1.00 |  |  |  |  |  |  |  |  |  |  |  |  |
| 23. Parental rejection T1 | **.10** | **-.31** | 1.00 |  |  |  |  |  |  |  |  |  |  |  |
| 24. Parental overprotection T1 | .01 | **.18** | **.43** | 1.00 |  |  |  |  |  |  |  |  |  |  |
| 25. Peer status T1 | .01 | **.17** | ***-.06*** | **.14** | 1.00 |  |  |  |  |  |  |  |  |  |
| 26. Peer affection T1 | **-.08** | **.37** | **-.20** | .04 | **.47** | 1.00 |  |  |  |  |  |  |  |  |
| 27. Family dysfunction T3 | **.45** | **-.12** | ***.07*** | .00 | .01 | -.03 | 1.00 |  |  |  |  |  |  |  |
| 28. Parental control T3 | *-.06* | **.16** | -.03 | **.09** | .04 | **.10** | -.04 | 1.00 |  |  |  |  |  |  |
| 29. Parental anger T3 | *.06* | .01 | **.19** | **.14** | -.01 | -.01 | **.12** | **.23** | 1.00 |  |  |  |  |  |
| 30. Parental guilt inducing T3 | ***.08*** | **-.10** | **.13** | **.09** | -.01 | -.04 | **.12** | -.02 | **.42** | 1.00 |  |  |  |  |
| 31. Parental problem solving T3 | **-.14** | **.31** | **-.14** | -.02 | .03 | **.16** | **-.12** | **.37** | -.04 | **-.23** | 1.00 |  |  |  |
| 32. Peer support T3 | -.03 | **.11** | **-.10** | -.01 | **.11** | **.15** | -.01 | ***.08*** | .00 | *-.07* | **.10** | 1.00 |  |  |
| 33. Practical help peers T3 | -.02 | ***.08*** | **-.11** | .00 | ***.07*** | ***.08*** | .00 | *.06* | -.03 | *-.07* | **.11** | **.50** | 1.00 |  |
| 34. Peer fights T3 | .05 | -.02 | ***.07*** | ***.07*** | .03 | .01 | .03 | .01 | **.18** | **.12** | .04 | .02 | -.02 | 1.00 |

*Note.* ^1^ 0 = female, 1 = male; ^2^ scores were reversed for analysis, thus a higher score indicates less negative functioning. Spearman correlations were computed for correlations with sex, happiness, satisfaction, positive affect, positive social functioning, personal achievement, daily occupation, affective problems, attention problems, antisocial personality problems, avoidant personality problems, parental rejection, parental guilt inducing, and peer fights. Correlations are based on existing data: *n* = 958 - 2230. Significant coefficients are indicated in italics (*p* < .05), bold and italics (*p* < .01), or bold (*p* < .001).

1. **Graphical overview final SEM models**

**
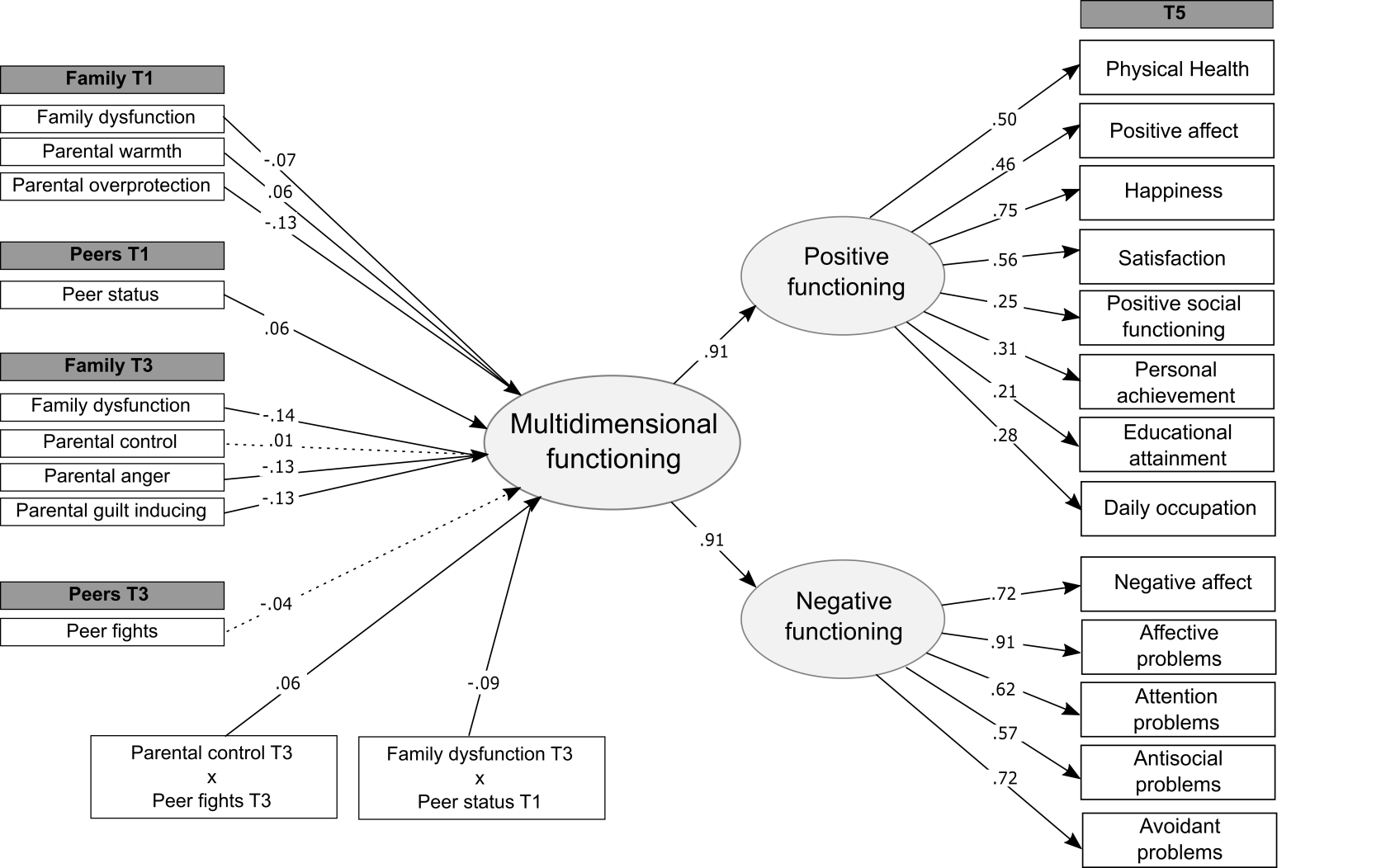
**

*Figure S1.* Graphical overview of the final SEM model predicting multidimensional functioning. Scores for negative functioning were reversed for analysis, thus a higher score indicates less negative functioning. For ease of interpretation covariances between independent variables and correlated residuals between variables measured by the same instrument were omitted (e.g., all variables measured by the Achenbach scales or the PANAS). The solid lines represent effects below *p* =.05; the dotted lines effects above *p* =.05.

**
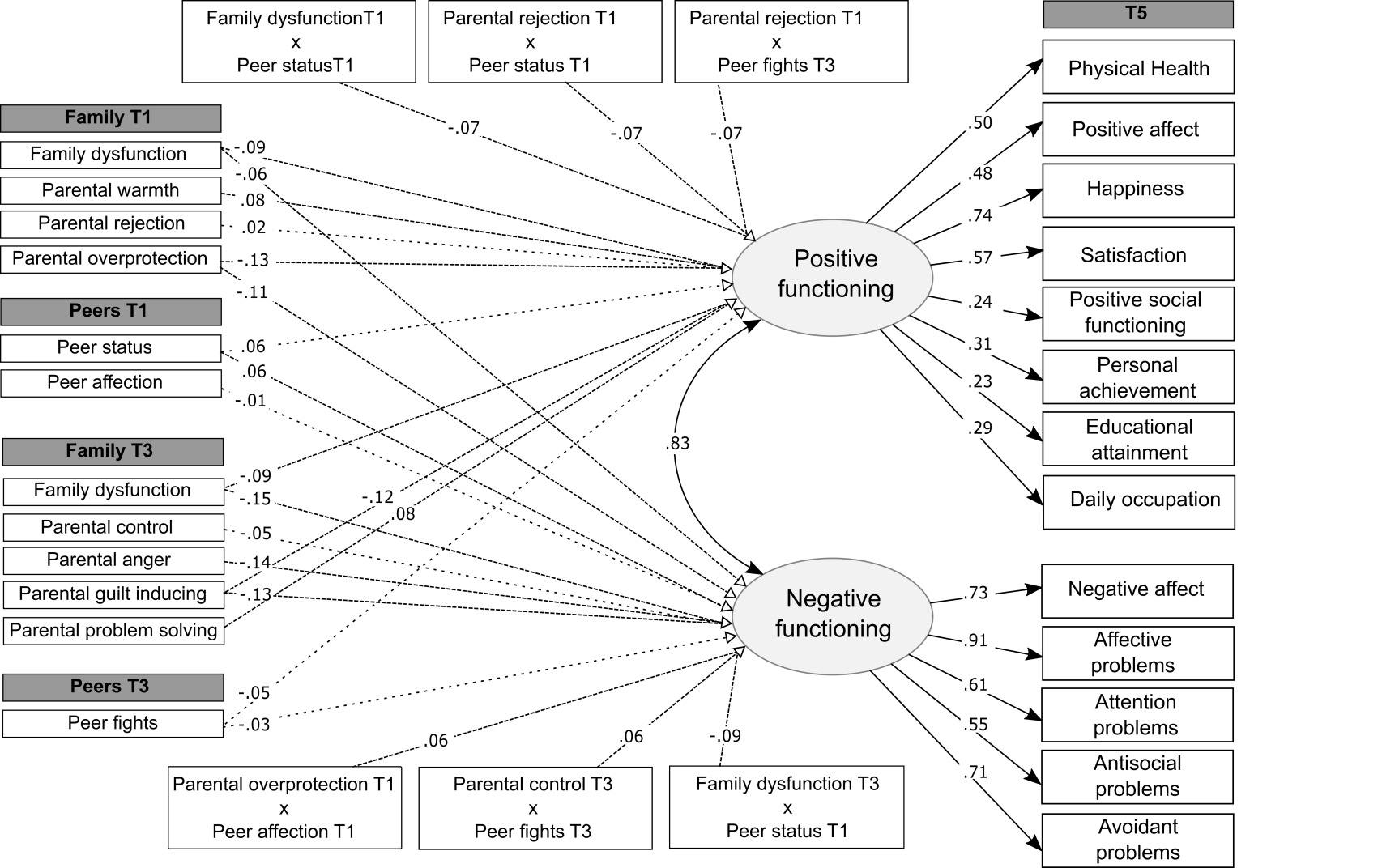
**

*Figure S2.* Graphical overview of the final SEM model predicting positive and negative functioning. Scores for negative functioning were reversed for analysis, thus a higher score indicates less negative functioning. For ease of interpretation covariances between independent variables and correlated residuals between variables measured by the same instrument were omitted (e.g., all variables measured by the Achenbach scales or the PANAS). The solid and dashed lines represent effects below *p* =.05; the dotted lines effects above *p* =.05.

**4. Sensitivity analyses**

Results of the final models controlling for sex, SES, number of parents, and mental health problems at age 11 are presented in Tables S6-S8.

**Table S6.** Results of structural equation models predicting young adult multidimensional functioning, controlling for sex, socio-economic status, number of parents at T1, and mental health problems at T1

|  | **Multidimensional functioning** | | | | | | | | | | | | | | |
| --- | --- | --- | --- | --- | --- | --- | --- | --- | --- | --- | --- | --- | --- | --- | --- |
|  | Model 1 | | | Model 2 | | | Model 3 | | | Model 4 | | | Model 5 | | |
|  | *β* | *SE* | *p* | *β* | *SE* | *p* | *β* | *SE* | *p* | *β* | *SE* | *p* | *β* | *SE* | *p* |
| *Controls* |  |  |  |  |  |  |  |  |  |  |  |  |  |  |  |
| Sex^1^ | **.16** | **.03** | **<.001** | **-** | **-** | **-** | **-** | **-** | **-** | **-** | **-** | **-** | **.16** | **.03** | **<.001** |
| SES | **-** | **-** | **-** | .03 | .03 | .372 | **-** | **-** | **-** | **-** | **-** | **-** | .00 | .03 | .971 |
| Number of parents | **-** | **-** | **-** | **-** | **-** | **-** | **-.39** | **.04** | **<.001** | **-** | **-** | **-** | .05 | .03 | .080 |
| MHPT1 | **-** | **-** | **-** | **-** | **-** | **-** | **-** | **-** | **-** | **-.39** | **.04** | **<.001** | **-.34** | **.03** | **<.001** |
| *Family T1* |  |  |  |  |  |  |  |  |  |  |  |  |  |  |  |
| Family dysfunction | -.07 | .03 | .020 | -.07 | .03 | .032 | *-.06* | *.03* | *.050* | *-.02* | *.04* | *.657* | *-.01* | *.03* | *.782* |
| Parental warmth | .07 | .03 | .017 | *.06* | *.03* | *.050* | .06 | .03 | .034 | *-.02* | *.03* | *.432* | *-.01* | *.03* | *.683* |
| Parental rejection | - | - | - | - | - | - | - | - | - | - | - | - | - | - | - |
| Parental overprotection | **-.15** | **.03** | **<.001** | **-.13** | **.03** | **<.001** | **-.14** | **.03** | **<.001** | *-.03* | *.03* | *.409* | *-.05* | *.03* | *.128* |
| *Peers T1* |  |  |  |  |  |  |  |  |  |  |  |  |  |  |  |
| Peer status | .07 | .03 | .016 | .06 | .03 | .043 | .06 | .03 | .045 | *.03* | *.03* | *.327* | *.04* | *.03* | *.205* |
| Peer affection | - | - | - | - | - | - | - | - | - | - | - | - | - | - | - |
| *Family T3* |  |  |  |  |  |  |  |  |  |  |  |  |  |  |  |
| Family dysfunction | **-.14** | **.04** | **<.001** | **-.14** | **.03** | **<.001** | **-.14** | **.04** | **<.001** | **-.15** | **.04** | **<.001** | **-.12** | **.04** | **<.001** |
| Parental control | .04 | .03 | .194 | .00 | .03 | .920 | .00 | .03 | .930 | -.01 | .03 | .716 | .02 | .03 | .570 |
| Parental anger | *-.11* | *.03* | *.001* | **-.13** | **.04** | **<.001** | **-.13** | **.03** | **<.001** | *-.12* | *.04* | *.001* | *-.08* | *.03* | *.008* |
| Parental guilt inducing | -.13 | .04 | .001 | -.13 | .04 | .001 | -.12 | .04 | .001 | **-.16** | **.04** | **<.001** | **-.13** | **.04** | **<.001** |
| Parental problem solving | - | - | - | - | - | - | - | - | - | - | - | - | - | - | - |
| *Peers T3* |  |  |  |  |  |  |  |  |  |  |  |  |  |  |  |
| Peer support | - | - | - | - | - | - | - | - | - | - | - | - | - | - | - |
| Practical help peers | - | - | - | - | - | - | - | - | - | - | - | - | - | - | - |
| Peer fights | -.02 | .03 | .553 | -.03 | .03 | .272 | -.04 | .03 | .232 | .00 | .03 | .999 | .02 | .03 | .586 |
|  |  |  |  |  |  |  |  |  |  |  |  |  |  |  |  |
| *Interaction effects* |  |  |  |  |  |  |  |  |  |  |  |  |  |  |  |
| Parental control T3 x Peer fights T3 | .06 | .03 | .041 | .06 | .03 | .037 | .06 | .03 | .037 | *.06* | *.03* | *.087* | *.05* | *.03* | *.077* |
| Family dysfunctionT3 x Peer status T1 | -.09 | .04 | .016 | -.09 | .04 | .018 | -.08 | .04 | .026 | -.09 | .04 | .013 | -.08 | .03 | .020 |
|  |  |  |  |  |  |  |  |  |  |  |  |  |  |  |  |
| *Model fit indices* |  |  |  |  |  |  |  |  |  |  |  |  |  |  |  |
| Chi^2^ | 1280.43 | |  | 1160.95 | |  | 870.62 | |  | 923.56 | |  | 1474.29 | |  |
| RMSEA | .05 |  |  | .05 |  |  | .04 |  |  | .04 |  |  | .05 |  |  |
| CFI | .85 |  |  | .86 |  |  | .90 |  |  | .89 |  |  | .84 |  |  |
| TLI | .82 |  |  | .83 |  |  | .88 |  |  | .87 |  |  | .80 |  |  |
| SRMR | .06 |  |  | .06 |  |  | .05 |  |  | .05 |  |  | .06 |  |  |
| R^2^ | .17 |  |  | .15 |  |  | .16 |  |  | .24 |  |  | .26 |  |  |

*Note*. ^1^ 0 = female, 1 = male; Structural equation models are based on maximum likelihood with robust standard error estimation (MLR), sample size *n* = 2228. Significant effects surviving the correction for multiple testing (*p* < .00066) are shown in bold. Effects that dropped in significance after controlling for sex, socio-economic status, number of parents at T1, and mental health problems at T1 are shown in italic.

**Table S7.** Results of structural equation models predicting young adult positive functioning, controlling for sex, socio-economic status, number of parents at T1, and mental health problems at T1

|  | **Positive functioning** | | | | | | | | | | | | | | |
| --- | --- | --- | --- | --- | --- | --- | --- | --- | --- | --- | --- | --- | --- | --- | --- |
|  | Model 1 | | | Model 2 | | | Model 3 | | | Model 4 | | | Model 5 | | |
|  | *β* | *SE* | *p* | *β* | *SE* | *p* | *β* | *SE* | *p* | *β* | *SE* | *p* | *β* | *SE* | *p* |
| *Controls* |  |  |  |  |  |  |  |  |  |  |  |  |  |  |  |
| Sex^1^ | .03 | .03 | .327 | **-** | **-** | **-** | **-** | **-** | **-** | **-** | **-** | **-** | .04 | .03 | .237 |
| SES | **-** | **-** | **-** | .08 | .05 | .080 | **-** | **-** | **-** | **-** | **-** | **-** | .06 | .05 | .178 |
| Number of parents | **-** | **-** | **-** | **-** | **-** | **-** | .06 | .03 | .086 | **-** | **-** | **-** | .03 | .03 | .366 |
| MHPT1 | **-** | **-** | **-** | **-** | **-** | **-** | **-** | **-** | **-** | **-.25** | **.04** | **<.001** | **-.24** | **.04** | **<.001** |
| *Family T1* |  |  |  |  |  |  |  |  |  |  |  |  |  |  |  |
| Family dysfunction | -.09 | .04 | .009 | -.09 | .04 | .016 | -.09 | .04 | .016 | *-.05* | *.04* | *.153* | *-.04* | *.04* | *.272* |
| Parental warmth | .08 | .03 | .012 | .08 | .03 | .016 | .08 | .03 | .013 | .08 | .03 | .022 | .07 | .03 | .026 |
| Parental rejection | .02 | .04 | .490 | .02 | .04 | .676 | .02 | .04 | .606 | .03 | .04 | .428 | .03 | .04 | .410 |
| Parental overprotection | **-.14** | **.04** | **<.001** | *-.12* | *.04* | *.001* | **-.14** | **.04** | **<.001** | *-.07* | *.04* | *.070* | *-.07* | *.04* | *.057* |
| *Peers T1* |  |  |  |  |  |  |  |  |  |  |  |  |  |  |  |
| Peer status | .07 | .04 | .061 | .06 | .04 | .092 | .06 | .04 | .079 | .03 | .03 | .396 | .03 | .04 | .393 |
| Peer affection | - | - | - | - | - | - | - | - | - | - | - | - | **-** | **-** | **-** |
| *Family T3* |  |  |  |  |  |  |  |  |  |  |  |  |  |  |  |
| Family dysfunction | -.09 | .04 | .026 | -.09 | .04 | .029 | -.09 | .04 | .024 | *-.07* | *.04* | *.066* | *-.07* | *.04* | *.082* |
| Parental control | - | - | - | - | - | - | - | - | - | - | - | - | **-** | **-** | **-** |
| Parental anger | - | - | - | - | - | - | - | - | - | - | - | - | **-** | **-** | **-** |
| Parental guilt inducing | -.11 | .04 | .002 | -.12 | .04 | .002 | -.11 | .04 | .003 | -.11 | .04 | .003 | -.11 | .04 | .004 |
| Parental problem solving | .08 | .03 | .005 | .08 | .03 | .007 | .08 | .03 | .005 | .08 | .03 | .005 | .08 | .03 | .010 |
| *Peers T3* |  |  |  |  |  |  |  |  |  |  |  |  |  |  |  |
| Peer support | - | - | - | - | - | - | - | - | - | - | - | - | **-** | **-** | **-** |
| Practical help peers | - | - | - | - | - | - | - | - | - | - | - | - | **-** | **-** | **-** |
| Peer fights | -.05 | .04 | .189 | -.05 | .04 | .140 | -.05 | .04 | .130 | -.03 | .04 | .446 | -.02 | .04 | .494 |
|  |  |  |  |  |  |  |  |  |  |  |  |  |  |  |  |
| *Interaction effects* |  |  |  |  |  |  |  |  |  |  |  |  |  |  |  |
| Family dysfunctionT1 x Peer status T1 | -.06 | .03 | .036 | -.07 | .03 | .030 | -.06 | .03 | .034 | -.06 | .03 | .034 | -.06 | .03 | .039 |
| Parental rejection T1 x Peer status T1 | -.06 | .03 | .034 | -.07 | .03 | .025 | .06 | .03 | .029 | -.07 | .03 | .018 | -.07 | .03 | .019 |
| Parental rejection T1 x Peer fights T3 | -.07 | .03 | .016 | -.07 | .03 | .025 | -.07 | .03 | .024 | -.07 | .03 | .030 | -.07 | .03 | .021 |
|  |  |  |  |  |  |  |  |  |  |  |  |  |  |  |  |
| *Model fit indices* |  |  |  |  |  |  |  |  |  |  |  |  |  |  |  |
| Chi^2^ | 1388.65 | |  | 1249.92 | |  | 965.20 | |  | 1010.02 | |  | 1509.06 | |  |
| RMSEA | .04 |  |  | .04 |  |  | .03 |  |  | .04 |  |  | .04 |  |  |
| CFI | .85 |  |  | .87 |  |  | .90 |  |  | .90 |  |  | .84 |  |  |
| TLI | .83 |  |  | .84 |  |  | .88 |  |  | .87 |  |  | .81 |  |  |
| SRMR | .05 |  |  | .05 |  |  | .04 |  |  | .04 |  |  | .05 |  |  |
| R^2^ | .11 |  |  | .13 |  |  | .12 |  |  | .17 |  |  | .18 |  |  |

*Note*. ^1^ 0 = female, 1 = male; SES = socio-economic status. Structural equation models are based on maximum likelihood with robust standard error estimation (MLR), sample size *n* = 2228. Significant effects surviving the correction for multiple testing (*p* < .00066) are shown in bold. Effects that dropped in significance after controlling for sex, socio-economic status, number of parents at T1, and mental health problems at T1 are shown in italic.

**Table S8.** Results of structural equation models predicting young adult negative functioning, controlling for sex, socio-economic status, number of parents at T1, and mental health problems at T1

|  | **Negative functioning** | | | | | | | | | | | | | | |
| --- | --- | --- | --- | --- | --- | --- | --- | --- | --- | --- | --- | --- | --- | --- | --- |
|  | Model 1 | | | Model 2 | | | Model 3 | | | Model 4 | | | Model 5 | | |
|  | *β* | *SE* | *p* | *β* | *SE* | *p* | *β* | *SE* | *p* | *β* | *SE* | *p* | *β* | *SE* | *p* |
| *Controls* |  |  |  |  |  |  |  |  |  |  |  |  |  |  |  |
| Sex^1^ | **-.19** | **.03** | **<.001** | **-** | **-** | **-** | **-** | **-** | **-** | **-** | **-** | **-** | **-.18** | **.03** | **<.001** |
| SES | **-** | **-** | **-** | -.02 | .03 | .538 | **-** | **-** | **-** | **-** | **-** | **-** | .02 | .03 | .395 |
| Number of parents | **-** | **-** | **-** | **-** | **-** | **-** | -.09 | .03 | .002 | **-** | **-** | **-** | -.06 | .03 | .036 |
| MHPT1 | **-** | **-** | **-** | **-** | **-** | **-** | **-** | **-** | **-** | **.32** | **.03** | **<.001** | **.32** | **.03** | **<.001** |
| *Family T1* |  |  |  |  |  |  |  |  |  |  |  |  |  |  |  |
| Family dysfunction | .06 | .00 | .042 | *.06* | *.03* | *.057* | *.05* | *.03* | *.090* | *.00* | *.03* | *.973* | *.00* | *.03* | *.935* |
| Parental warmth | - | - | - | - | - | - | - | - | - | - | - | - | **-** | **-** | **-** |
| Parental rejection | - | - | - | - | - | - | - | - | - | - | - | - | **-** | **-** | **-** |
| Parental overprotection | **.13** | **.03** | **<.001** | **.11** | **.03** | **<.001** | **.12** | **.03** | **<.001** | *.02* | *.03* | *.400* | *-.04* | *.03* | *.102* |
| *Peers T1* |  |  |  |  |  |  |  |  |  |  |  |  |  |  |  |
| Peer status | -.06 | .03 | .023 | -.06 | .03 | .033 | -.06 | .03 | .039 | *-.04* | *.03* | *.148* | *-.04* | *.03* | *.122* |
| Peer affection | -.01 | .02 | .695 | .01 | .02 | .593 | .01 | .02 | .690 | .05 | .02 | .023 | .03 | .02 | .138 |
| *Family T3* |  |  |  |  |  |  |  |  |  |  |  |  |  |  |  |
| Family dysfunction | **.14** | **.03** | **<.001** | **.15** | **.03** | **<.001** | **.15** | **.03** | **<.001** | **.13** | **.03** | **<.001** | **.12** | **.03** | **<.001** |
| Parental control | .02 | .02 | .538 | .05 | .02 | .069 | .05 | .02 | .032 | .05 | .02 | .020 | .03 | .02 | .255 |
| Parental anger | **.11** | **.03** | **<.001** | **.14** | **.03** | **<.001** | **.14** | **.03** | **<.001** | **.12** | **.02** | **<.001** | **.09** | **.02** | **<.001** |
| Parental guilt inducing | **.12** | **.04** | **<.001** | **.13** | **.04** | **<.001** | *.12* | *.04* | *.001* | **.12** | **.04** | **<.001** | *.12* | *.03* | *.001* |
| Parental problem solving | - | - | - | - | - | - | - | - | - | - | - | - | **-** | **-** | **-** |
| *Peers T3* |  |  |  |  |  |  |  |  |  |  |  |  |  |  |  |
| Peer support | - | - | - | - | - | - | - | - | - | - | - | - | **-** | **-** | **-** |
| Practical help peers | - | - | - | - | - | - | - | - | - | - | - | - | **-** | **-** | **-** |
| Peer fights | .01 | .03 | .640 | .03 | .03 | .269 | .04 | .03 | .230 | .00 | .03 | .957 | -.02 | .03 | .490 |
|  |  |  |  |  |  |  |  |  |  |  |  |  |  |  |  |
| *Interaction effects* |  |  |  |  |  |  |  |  |  |  |  |  |  |  |  |
| Parental overprotection T1 x Peer affection T1 | -.06 | .02 | .011 | -.06 | .02 | .007 | -.06 | .02 | .008 | -.05 | .02 | .027 | -.04 | .02 | .038 |
| Parental control T3 x Peer fights T3 | -.06 | .02 | .004 | -.06 | .02 | .005 | -.06 | .02 | .004 | -.05 | .02 | .027 | -.05 | .02 | .013 |
| Family dysfunctionT3 x Peer status T1 | .09 | .03 | .002 | .09 | .03 | .002 | .08 | .03 | .004 | .08 | .03 | .003 | .08 | .03 | .002 |
|  |  |  |  |  |  |  |  |  |  |  |  |  |  |  |  |
| *Model fit indices* |  |  |  |  |  |  |  |  |  |  |  |  |  |  |  |
| Chi^2^ | 1388.646 | |  | 1249.919 | |  | 695.20 | |  | 1010.021 | |  | 1509.06 | |  |
| RMSEA | .04 |  |  | .04 |  |  | .03 |  |  | .04 |  |  | .04 |  |  |
| CFI | .85 |  |  | .87 |  |  | .90 |  |  | .90 |  |  | .84 |  |  |
| TLI | .83 |  |  | .84 |  |  | .88 |  |  | .87 |  |  | .81 |  |  |
| SRMR | .05 |  |  | .05 |  |  | .04 |  |  | .04 |  |  | .05 |  |  |
| R^2^ | .16 |  |  | .14 |  |  | .15 |  |  | .23 |  |  | .25 |  |  |

*Note*. ^1^ 0 = female, 1 = male; Structural equation models are based on maximum likelihood with robust standard error estimation (MLR), sample size *n* = 2228. Significant effects surviving the correction for multiple testing (*p* < .00066) are shown in bold. Effects that dropped in significance after controlling for sex, socio-economic status, number of parents at T1, and mental health problems at T1 are shown in italic.

**References**

**Achenbach T, Rescorla L** (2001). ASEBA school-age forms & profiles.

Burlington, VT: University of Vermont.

**Achenbach T, Rescorla L** (2003). *Manual for ASEBA Adult Forms & Profiles*. Burlington, VT: University of Vermont.

**Crawford JR, Henry JD** (2004). The Positive and Negative Affect Schedule (PANAS): Construct validity, measurement properties and normative data in a large non-clinical sample. *British Journal of Clinical Psychology* **43**, 245–265.

**Epstein NB, Baldwin LM, Bishop DS** (1983). The McMAster Family Assessment Device. *Journal of Marital and Family Therapy* **9**, 171–180.

**Ganzeboom H, Treiman DJ** (1996). Internationally comparable measures of occupational status for the 1988 International Standard Classification of Occupations. *Social Science Research* ***25,*** *201-239*

**Kristensen TS, Hannerz H, Høgh A, Borg V** (2005). The Copenhagen Psychosocial Questionnaire - a tool for the assessment and improvement of the psychosocial work environment. *Scandinavian journal of Work, Environment & Health* **31**, 438–49.

**Li J & Ji L** (2005). Adjusting multiple testing in multilocus analyses using the eigenvalues of a correlation matrix. *Heredity* **95**, 221–227.

**MacKinnon A, Jorm A, Christensen H, Korten A, Jacomb P** (1999). A short form of the Positive and Negative Affect Schedule: Evaluation of factorial validity and invariance across demographic variables in a community sample. *Personality and Individual Differences* **27**, 405–416.

**Markus M, Lindhout I, Boer F, Hoogendijk T, Arrindell W** (2003). Factors of perceived parental rearing styles: the EMBU-C examined in a sample of Dutch primary school children. *Personality and Individual Differences* **34**, 503–519.

**Nieboer A, Lindenberg S, Boomsma A, Bruggen A** (2005). Dimensions Of Well-Being And Their Measurement: The SPF-Il Scale. *Social Indicators Research* **73**, 313–353.

**Ormel J, Lindenberg S, Steverink N, Vonkorff M** (1997). Quality of life and social production functions: a framework for understanding health effects. *Social science & medicine (1982)* **45**, 1051–63.

**Poulin F, Pedersen S** (2007). Developmental changes in gender composition of friendship networks in adolescent girls and boys. *Developmental Psychology* **43**, 1484.

**Rusbult CE, John M, Agnew CR** (1998). The Investment Model Scale: Measuring commitment level, satisfaction level, quality of alternatives, and investment size. *Personal Relationships* **5**, 357–387.

**Stattin H, Kerr M** (2000). Parental Monitoring: A Reinterpretation. *Child Development* **71**, 1072–1085.

**Tilton-Weaver L, Kerr M, Pakalniskeine V, Tokic A, Salihovic S, Stattin H** (2010). Open up or close down: How do parental reactions affect youth information management? *Journal of Adolescence* **33**, 333–346.

**Veldman K, Bültmann U, Stewart R, Ormel J, Verhulst F, Reijneveld S** (2014). Mental Health Problems and Educational Attainment in Adolescence: 9-Year Follow-Up of the TRAILS Study. *PLoS ONE* **9**, e101751.

**Verhulst F, Ende J** (1992). Agreement Between Parents’ Reports and Adolescents' Self-reports of Problem Behavior. *Journal of Child Psychology and Psychiatry* **33**, 1011–1023.

**Watson D, Clark L, Tellegen A** (1988). Development and validation of brief measures of positive and negative affect: The PANAS scales. *Journal of Personality and Social Psychology* **54**, 1063.
